# Association between age and the host response in critically ill patients with sepsis

**DOI:** 10.1186/s13054-022-04266-9

**Published:** 2022-12-13

**Authors:** Erik H. A. Michels, Joe M. Butler, Tom D. Y. Reijnders, Olaf L. Cremer, Brendon P. Scicluna, Fabrice Uhel, Hessel Peters-Sengers, Marcus J. Schultz, Julian C. Knight, Lonneke A. van Vught, Tom van der Poll, MARS consortium, Friso M. de Beer, Lieuwe D. J. Bos, Gerie J. Glas, Arie J. Hoogendijk, Roosmarijn T. M. van Hooijdonk, Janneke Horn, Mischa A. Huson, Laura R. A. Schouten, Marleen Straat, Luuk Wieske, Maryse A. Wiewel, Esther Witteveen, Marc J. M. Bonten, Olaf M. Cremer, David S. Y. Ong, Jos F. Frencken, Peter M. C. Klein Klouwenberg, Maria E. Koster‐Brouwer, Kirsten van de Groep, Diana M. Verboom

**Affiliations:** 1 Center of Experimental and Molecular Medicine, Amsterdam University Medical Centers, University of Amsterdam, Meibergdreef 9, 1105AZ Amsterdam, The Netherlands; 2 Department of Intensive Care Medicine, University Medical Center Utrecht, Utrecht, The Netherlands; 3 Department of Applied Biomedical Science, Faculty of Health Sciences, Mater Dei Hospital, University of Malta, Msida, Malta; 4 Centre for Molecular Medicine and Biobanking, University of Malta, Msida, Malta; 5 Department of Intensive Care, Amsterdam University Medical Centers, University of Amsterdam, Amsterdam, The Netherlands; 6 Mahidol-Oxford Tropical Medicine Research Unit (MORU), Mahidol University, Bangkok, Thailand; 7 Nuffield Department of Medicine, University of Oxford, Oxford, UK; 8 Wellcome Centre for Human Genetics, University of Oxford, Oxford, UK; 9 Division of Infectious Diseases, Amsterdam UMC Location University of Amsterdam, Amsterdam, the Netherlands

**Keywords:** Ageing, Sepsis, Immune system, Biomarkers, Coagulation, Endothelium, Cytokines, Inflammation, Transcriptome

## Abstract

**Background:**

The association of ageing with increased sepsis mortality is well established. Nonetheless, current investigations on the influence of age on host response aberrations are largely limited to plasma cytokine levels while neglecting other pathophysiological sepsis domains like endothelial cell activation and function, and coagulation activation. The primary objective of this study was to gain insight into the association of ageing with aberrations in key host response pathways and blood transcriptomes in sepsis.

**Methods:**

We analysed the clinical outcome (*n* = 1952), 16 plasma biomarkers providing insight in deregulation of specific pathophysiological domains (*n* = 899), and blood leukocyte transcriptomes (*n* = 488) of sepsis patients stratified according to age decades. Blood transcriptome results were validated in an independent sepsis cohort and compared with healthy individuals.

**Results:**

Older age was associated with increased mortality independent of comorbidities and disease severity. Ageing was associated with lower endothelial cell activation and dysfunction, and similar inflammation and coagulation activation, despite higher disease severity scores. Blood leukocytes of patients ≥ 70 years, compared to patients < 50 years, showed decreased expression of genes involved in cytokine signaling, and innate and adaptive immunity, and increased expression of genes involved in hemostasis and endothelial cell activation. The diminished expression of gene pathways related to innate immunity and cytokine signaling in subjects ≥ 70 years was sepsis-induced, as healthy subjects ≥ 70 years showed enhanced expression of these pathways compared to healthy individuals < 50 years.

**Conclusions:**

This study provides novel evidence that older age is associated with relatively mitigated sepsis-induced endothelial cell activation and dysfunction, and a blood leukocyte transcriptome signature indicating impaired innate immune and cytokine signaling. These data suggest that age should be considered in patient selection in future sepsis trials targeting the immune system and/or the endothelial cell response.

**Graphical abstract:**

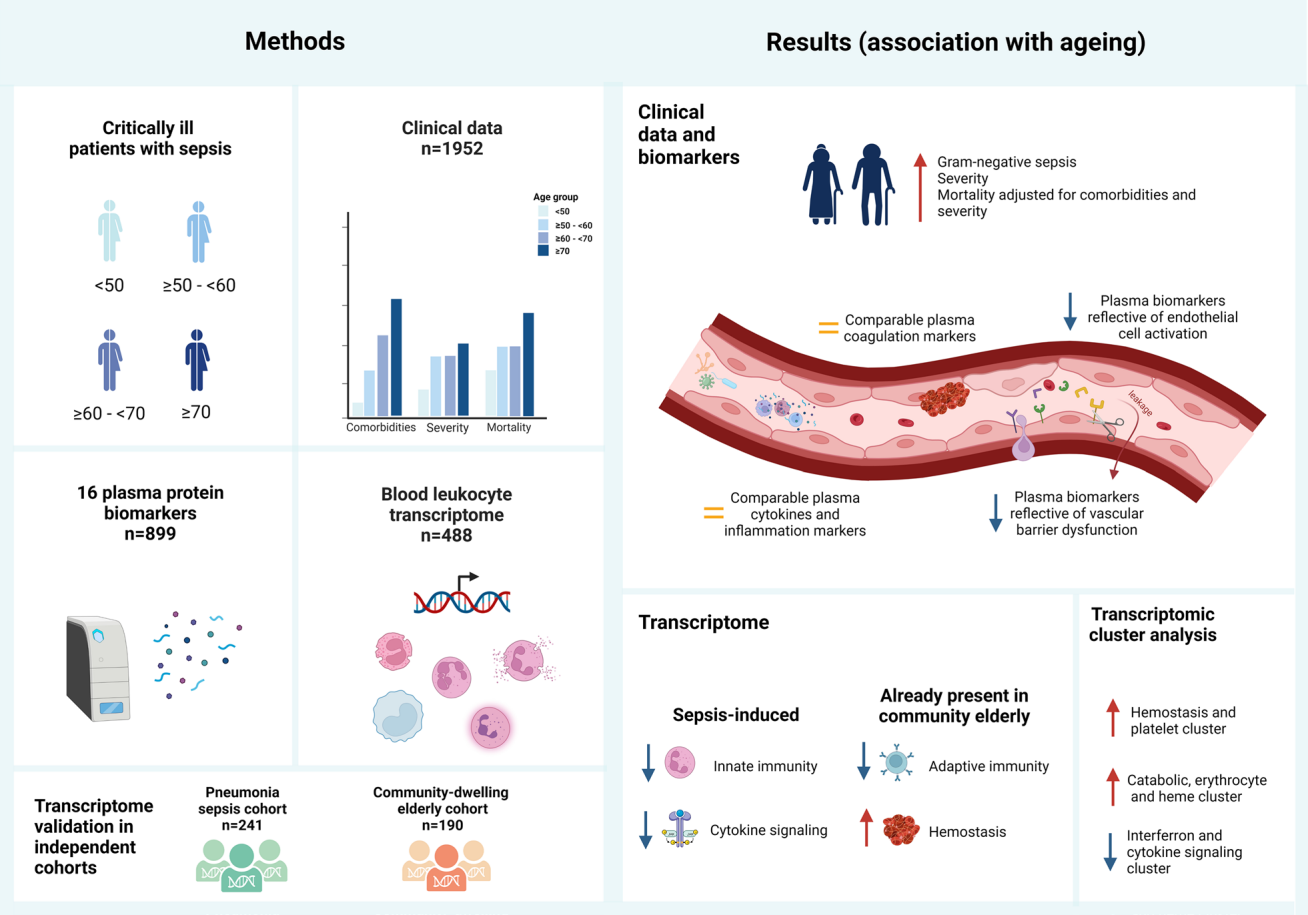

**Supplementary Information:**

The online version contains supplementary material available at 10.1186/s13054-022-04266-9.

## Introduction

Sepsis is defined as a dysregulated host response to an infection leading to organ failure [1]. Sepsis is the leading cause of in-hospital mortality and accounts for an estimated 19.7% of all global deaths [2]. The incidence of sepsis and sepsis-related mortality increase dramatically with old age [3–7]. Individuals aged 65 and older comprise ~ 65% of the sepsis cases in the United States while accounting for only 12% of the population [3]. This increase in incidence and mortality can partly be explained by age-related comorbidities [3, 5]. Yet, extensive research has shown that age-related changes in the immune system, named immunosenescence, may be an additional important factor [8–12]. Immunosenescence refers to the age-related (functional) changes in various innate and adaptive immune cells observed in primarily in vitro and animal experiments [8, 9]. A notable example of immunosenescence is the decreasing ability of elderly persons to mount an effective immune response to new antigens [13]. In contrast, during a non-infectious state, “Inflammageing” is a well-described age-related immune phenomenon [14–16]. Inflammaging entails the sustained low-grade inflammation observed in the community-dwelling older adults [14–16]. Despite the known impact of age on clinical outcomes in sepsis, our understanding of how ageing influences the host response during sepsis remains incomplete. Previous studies in sepsis and community-acquired pneumonia (CAP) focused on plasma cytokines and other inflammation markers [17–24]. While most studies reported little to no association between age and inflammatory markers [18–24], one investigation found higher concentrations among older sepsis patients [17]. One study reported an association between increasing age and an enhanced coagulation response in patients with CAP [22]. Host response pathways implicated in the pathogenesis of sepsis—such as endothelial cell activation and dysfunction, and activation of the coagulation system [25–27]—have not been studied in the context of sepsis and ageing in patients.

The primary objective of this study was to gain insight into the association between age and aberrations in key host response pathways in sepsis. To this end, we measured 16 host response biomarkers indicative of alterations in three pathophysiological domains: systemic inflammation and cytokine release, endothelial cell activation and function, and coagulation activation in critically ill patients with sepsis stratified according to age. Additionally, we sought to determine the influence of age on the expression of genes in blood leukocytes involved in these three pathophysiological domains.

## Methods

### Study design and population

The Molecular Diagnosis and Risk Stratification of Sepsis (MARS) project (ClinicalTrials.gov identifier NCT0195033) was a prospective observational study in two tertiary hospitals in the Netherlands (January 2011–January 2014). Inclusion of patients was done as described [28]. For the current analysis, consecutive patients with sepsis diagnosed within 24 h after intensive care (ICU) admission were selected. Only the first admission for sepsis was included. Transfers were excluded. Sepsis was defined according to the Sepsis-3 criteria [1]; patients were post hoc labelled as fulfilling the Sepsis-3 criteria based on data prospectively collected during the inclusion period. The likelihood of infections was classified as “none”, “possible”, “probable”, or “definite” as described [28]. Definitions of comorbidities and outcomes are listed in the supplementary methods (Additional file 1). We also analysed transcriptomic data of two publicly available independent cohorts: a cohort of community-dwelling elderly in the Netherlands and a CAP sepsis cohort [29, 30].

### Measurements

For plasma biomarker assays and the analysis of whole blood leukocyte transcriptomes (using Affymetrix Human Genome U219 arrays) please see the supplementary methods in Additional file 1.

### Statistical and bioinformatics analysis

For specific details, please see the supplementary methods (Additional file 1). Patients were stratified according to age decades: < 50, ≥ 50–< 60, ≥ 60–< 70, and ≥ 70 years. This method was chosen to facilitate clinical interpretation and improve comparability. The protein biomarker and transcriptomic analysis was limited to patients with a probable or definite infection likelihood [28]. The association of ageing with all biomarkers was analysed using an analysis of variance (ANOVA) followed by a Tukey post hoc test if significant. The dependency of the results on stratification in age decades was analysed using a sensitivity analysis in which age was modeled as a continuous variable. Prior research has shown that ageing is associated with increased sepsis severity [3]. Moreover, the release of host response biomarkers in sepsis is often proportional to the severity of the disease [31]. Therefore, we performed a secondary analysis in which we sought to evaluate if ageing-associated host response aberrations change when correcting for the ageing-driven higher disease severity. All reported variables demonstrated < 5% missingness, except for C-reactive protein (27% missing) which demonstrated no patterns and was therefore classified as missing at random.

To assess differences in gene expression, patients ≥ 70 were compared to patients < 50 by performing BH adjusted moderated t-statistics using limma [32]. For the gene set enrichment analysis, Reactome pathways were selected to reflect the same three sepsis domains as the biomarker analysis [33]. The Benjamini–Hochberg (BH) adjustment was applied to the p-values of all 2387 available Reactome pathways [33]. We sought to externally validate the results of our pathway analysis (both direction and significance) by repeating the same analysis in an independent CAP cohort [30]. We also replicated the analysis in a cohort of healthy Dutch individuals to evaluate if differences were sepsis-specific or already present in healthy individuals [29]. Next, we performed a weighted gene co-expression network analysis (WGCNA) incorporating all ages rather than comparing extremes [34]. Modules were identified using the dynamic tree cut algorithm [34]. To label modules, each module was assigned a random color. Modules with a correlation coefficient greater than 0.75 were merged [35]. Genes weakly correlated to all modules (|eigengene-based connectivity|< 0.7) were not assigned [36, 37]. Relevant modules were identified based on the average absolute gene significance of all genes in a module and significant differences in the module eigengene between patients < 50 and ≥ 70. Functional profiling was conducted in g:Profiler [38]. Hub genes were identified by the Maximal Clique Centrality algorithm (CytoHubba) [39]. A detailed explanation of the WGCNA is provided in the supplementary methods.

## Results

### Patients

During the 3-year study period, 2785 ICU admissions for sepsis occurred (Additional file 2: Fig. S1). After exclusion of transfers from other ICUs (*n* = 296) and readmissions (*n* = 537), 1952 unique patients with sepsis remained. Of these, 421 patients (22%) were younger than 50 years at ICU admission, 368 (19%) ≥ 50–< 60 years, 545 (28%), ≥ 60–< 70 years, and 618 (32%) ≥ 70 years (Table 1). The proportion of males was lower in patients < 50 years; in this age group, the percentages of a white race and body mass index were lowest. The primary site of infection was broadly similar between age groups, except for central nervous system infections, which were more frequent in patients < 50 years (Table 1 and Additional file 1: Table S1). Gram-negative bacteria were less likely to be the causative pathogen in patients < 50 years, while viral infections were more common in this age group. The proportion of septic shock and acute kidney injury on ICU admission was lowest in patients < 50 and highest in patients ≥ 70 years.

**Table 1:** Baseline characteristics of critically ill sepsis patients stratified by age decade

	< 50 years	≥ 50–< 60 years	≥ 60–< 70 years	≥ 70 years	*p* value
n	421	368	545	618	
*Demographics*					
Age years, median [IQR]	39.00 [30.00, 45.00]*^ab^	55.00 [53.00, 57.00]*^a^	65.00 [62.00, 67.00]*	76.00 [72.00, 80.00]	< 0.001
Male sex, *n* (%)	234 (55.6)*^b^	251 (68.2)	339 (62.2)	394 (63.8)	0.003
White race, *n* (%)	336 (80.0)*^ab^	331 (89.9)*	499 (92.1)	584 (95.0)	< 0.001
BMI, median [IQR]	23.71 [21.51, 27.16]*^ab^	24.69 [21.61, 28.05]*^a^	25.71 [22.84, 29.39]	25.42 [22.86, 28.40]	< 0.001
Medical admission, *n* (%)	307 (72.9)	277 (75.3)	408 (74.9)	452 (73.1)	0.799
*Comorbidity*					
Charlson score^c^, median [IQR]	0.00 [0.00, 2.00]*^ab^	1.00 [0.00, 3.00] *^a^	2.00 [0.00, 3.00]	2.00 [1.00, 3.00]	< 0.001
Cardiovascular, *n* (%)	46 (10.9)*^ab^	76 (20.7)*^a^	163 (29.9)*	227 (36.7)	< 0.001
Respiratory insufficiency, *n* (%)	24 (5.7)*^a^	22 (6.0) *^a^	52 (9.5)	59 (9.5)	0.032
Hypertension, *n* (%)	57 (13.5)*^ab^	85 (23.1) *^a^	193 (35.4)*	268 (43.4)	< 0.001
Diabetes, *n* (%)	43 (10.2)*^a^	56 (15.2)*^a^	128 (23.5)	160 (25.9)	< 0.001
Malignancy, *n* (%)	71 (16.9)*^ab^	97 (26.4)	155 (28.4)	148 (23.9)	< 0.001
Renal disease, *n* (%)	38 (9.0)*^ab^	55 (14.9)	83 (15.2)	89 (14.4)	0.022
Immunocompromised, *n* (%)	114 (27.1)*	92 (25.1)*	136 (25.0)*	96 (15.6)	< 0.001
*Chronic medication, n (%)*					
Anticoagulants	34 (8.1)*^a^	38 (10.4)*^a^	98 (18.0)	131 (21.2)	< 0.001
Antiplatelet drugs	23 (5.7)*^ab^	62 (17.4)*^a^	139 (26.5)*	254 (42.2)	< 0.001
*Site of infection, n (%)*					
Cardiovascular	18 (4.3)	8 (2.2)	19 (3.5)	20 (3.2)	0.431
Pulmonary	193 (45.8)	160 (43.5)	232 (42.6)	280 (45.3)	0.700
Urinary	20 (4.8)	13 (3.5)	37 (6.8)	34 (5.5)	0.174
Skin	19 (4.5)	17 (4.6)	14 (2.6)	23 (3.7)	0.312
Abdominal	48 (11.4)	56 (15.2)	78 (14.3)	94 (15.2)	0.314
Central nervous system	32 (7.6)*^ab^	13 (3.5)	19 (3.5)	10 (1.6)	< 0.001
Other infection site^d^	15 (3.6)	19 (5.2)	33 (6.1)	27 (4.4)	0.304
Mixed infection	70 (16.6)	79 (21.5)	103 (18.9)	114 (18.4)	0.380
Unknown site **^b^	15 (3.6)	14 (3.8)	21 (3.9)	31 (5.0)	0.623
*Causative pathogen primary site of infection*^*e*^*, n (%)*					
Gram-positive bacteria	150 (35.8)	121 (33.2)	187 (34.4)	201 (32.8)	0.764
Gram-negative bacteria	111 (26.5)*^a^	117 (32.1)	189 (34.7)	209 (34.1)	0.031
Fungi	31 (7.4)	35 (9.6)	53 (9.7)*	34 (5.5)	0.032
Virus	28 (6.7)*	17 (4.7)	29 (5.3)	16 (2.6)	0.017
Other	18 (4.3)	6 (1.6)	17 (3.1)	15 (2.4)	0.138
Unknown	145 (34.6)	130 (35.6)	176 (32.4)	225 (36.7)	0.470
*Disease severity on admission*					
APACHE IV APS, median [IQR]	63.00 [47.00, 81.00]	67.00 [49.00, 84.25]	65.00 [50.00, 86.00]	67.00 [51.00, 85.00]	0.140
SOFA score^f^, median [IQR]	6.00 [3.00, 9.00]*^ab^	7.00 [4.00, 9.00]	7.00 [4.00, 9.00]	7.00 [5.00, 9.00]	0.001
Shock, *n* (%)	71 (16.9)*^b^	93 (25.3)	117 (21.5)*	175 (28.3)	< 0.001
ARDS, *n* (%)	93 (22.1)	89 (24.2)	113 (20.7)	126 (20.4)	0.514
Acute kidney injury, *n* (%)	110 (26.1)*	121 (32.9)	167 (30.6)*	237 (38.3)	< 0.001

Pairwise comparisons were made using the Dunn's Test of Multiple Comparisons Using Rank Sums followed by the Benjamini–Hochberg correction for multiple testing

*BMI* Body Mass Index, *APACHE IV APS* Acute Physiology and Chronic Health Evaluation IV Acute Physiology score, *SOFA* Sequential Organ Failure Score, *ARDS* acute respiratory distress syndrome

*p < 0.05 compared to the ≥ 70 group

^a^*p* < 0.05 compared to the ≥ 60–< 70 years group

^b^*p* < 0.05 compared to the ≥ 50–< 60 years group

^c^The Charlson score was calculated without the age component

^d^Other infection sites consisted of infections of bones and joints, the reproductive tract, mediastinum, the ear, throat or mouth. Specific sites per age group are shown in Additional file 1: Table S1

^e^Causative organisms of the primary site of infection do not add up to 100% as some patients suffered from multiple pathogens at the primary site

^f^The SOFA score was calculated without the Central Nervous System component

**Unknown site of infection consisted of infections of unknown source, systemic viral infections, and primary bacteremia. Specific sites per age group are shown in Additional file 1: Table S1

### Outcome

**Fig. 1:**
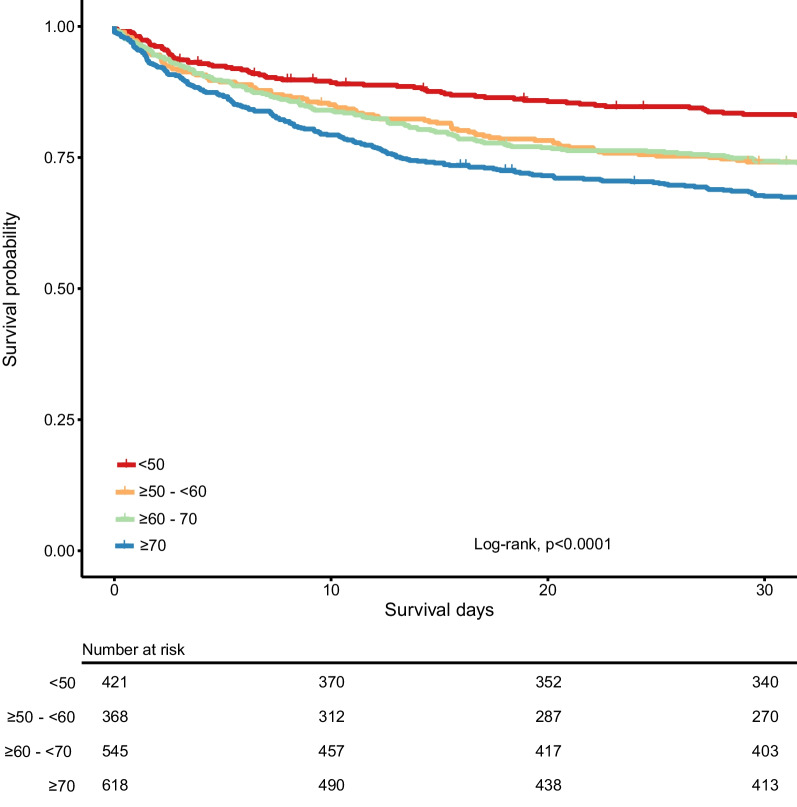
Thirty-day Kaplan–Meier survival curve of critically ill sepsis patients stratified by age decades

Lengths of stay and ICU-acquired complications did not differ between age groups (Table 2). As expected, short- and long-term mortality was highest in patients ≥ 70 and lowest in < 50 years (Table 2, Fig. 1). An increase in age group was associated with an increased risk for 30-day mortality, which was independent of differences in demographics, age-related comorbidities, the prevalence of anticoagulants or antiplatelet drugs, and baseline disease severity (Table 3).

**Table 2:** Clinical outcome of critically ill sepsis patients stratified by age decade

	< 50 years	≥ 50—< 60 years	≥ 60—< 70 years	≥ 70 years	*P* value
*n*	421	368	545	618	
*ICU-acquired complications, n (%)*					
Shock	52 (12.4)	43 (11.7)	89 (16.3)	78 (12.6)	0.128
ARDS	13 (3.1)	19 (5.2)	21 (3.9)	22 (3.6)	0.472
Acute kidney injury	25 (5.9)	22 (6.0)	50 (9.2)	42 (6.8)	0.158
ICU-acquired infections	40 (9.5)	33 (9.0)	45 (8.3)	54 (8.7)	0.925
*Length of stay, median [IQR]*					
ICU stay, days^c^	3.48 [1.63, 8.49]	4.63 [2.09, 9.35]	3.98 [1.82, 8.50]	3.84 [1.80, 7.79]	0.128
Hospital stay, days^d^	18.26 [8.20, 41.80]	22.10 [12.38, 38.38]	19.32 [10.84, 37.75]	18.24 [10.93, 33.17]	0.183
*Mortality, n (%)*					
ICU	54 (12.8)*	70 (19.0)	98 (18.0)	130 (21.0)	0.008
Hospital	80 (19.0)*^ab^	106 (28.9)	168 (31.1)	206 (33.3)	< 0.001
Day 30	81 (19.2)*^ab^	98 (26.7)*	142 (26.2)*	205 (33.2)	< 0.001
Day 60	99 (23.5)*^a^	110 (30.0)*	182 (33.6)	243 (39.3)	< 0.001
Day 90	112 (26.6)*^ab^	125 (34.1)*	203 (37.5)	265 (42.9)	< 0.001

Pairwise comparisons were made using the Dunn's Test of Multiple Comparisons Using Rank Sums followed by the Benjamini–Hochberg correction for multiple testing

*ARDS* acute respiratory distress syndrome, *ICU* intensive care

*p < 0.05 compared to the ≥ 70 group

^a^*p* < 0.05 compared to the ≥ 60–< 70 years group

^b^*p* < 0.05 compared to the ≥ 50–< 60 years group

^c^Length of ICU stay was only calculated in those who survived the entire ICU admission

^d^Length of hospital was only calculated in those who survived the entire hospital admission

**Table 3:** Adjusted and unadjusted 30-day mortality results of a Cox proportional hazard model

	Hazard ratio with 95% confidence interval			
Age group	Unadjusted	*p* value	Adjusted*	*p* value
< 50	1.0 (ref)		1.0 (ref)	
≥ 50–< 60 years	1.44 [1.07–1.93]	*p* < 0.05	1.35 [0.99–1.85]	*p* = 0.05
≥ 60–< 70 years	1.41 [1.08–1.87]	*p* < 0.05	1.46 [1.09–2.00]	*p* < 0.05
≥ 70 years	1.89 [1.46–2.45]	*p* < 0.001	2.11 [1.57–2.84]	*p* < 0.001

*Ref* reference category

*The adjusted model included demographics (BMI, sex, race, the inclusion hospital), ageing-associated comorbidities (chronic cardiovascular or pulmonary disease, hypertension, diabetes, malignancy, renal disease, Immunocompromised), chronic use of anticoagulant or antiplatelet drugs, disease severity on admission (SOFA, APACHE IV APS score, shock and acute kidney injury on admission)

### Host response biomarkers

**Fig. 2:**
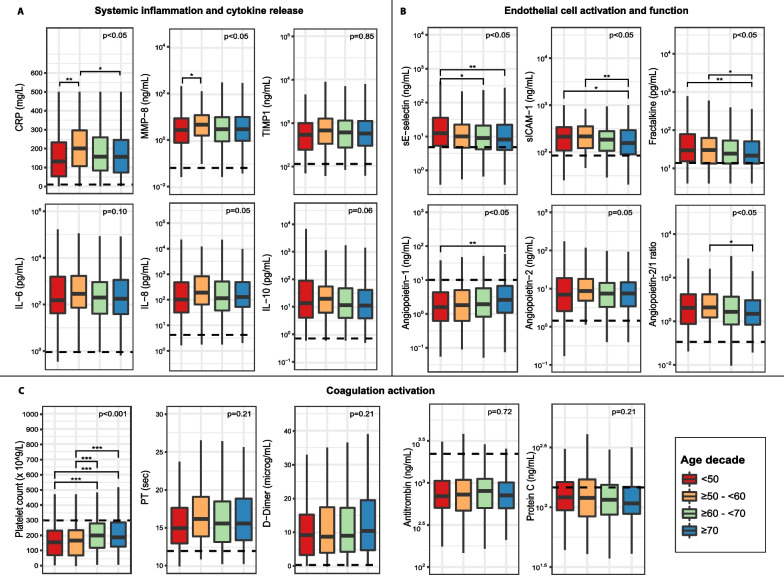
Host response biomarkers of critically ill sepsis patients stratified by age decades. Data of 889 patients expressed as box-and-whisker diagrams with whiskers ranging up to 1.5 times the interquartile range in which each panel reflects a host response domain. **A** Systemic inflammation and cytokine release markers. **B** Endothelial cell activation and function markers. **C** Coagulation activation markers. The dotted line represents the median concentration measured in 27 healthy controls. *P* values were derived form an ANOVA comparing the biomarker concentration between age decades. All p-values are multiple testing corrected using the Benjamini–Hochberg procedure for conducting 17 ANOVA’s (testing 17 biomarkers). Pairwise comparisons using a Dunn’s test were also multiple testing corrected. * *p* < 0.05, ** *p* < 0.01, *** *p* < 0.001. Abbreviations: ANOVA: analysis of variance, adj: adjusted, CRP: C-reactive protein, MMP-8: matrix metalloproteinase-8, TIMP-1: tissue inhibitor of metalloproteinase-1, IL-6: interleukin 6, IL-8: interleukin 8, IL-10: interleukin 10, sE-selectin: soluble E-Selectin, sICAM: soluble Intercellular Adhesion Molecule 1, PT: prothrombin time

Biomarkers were measured in the subgroup of patients with sepsis enrolled during the first 2.5 years with an infection likelihood of definite or probable (*n* = 889, Additional file 1: Tables S2 and S3) [28]. Sixteen host response biomarkers were determined to provide insight into physiological pathways implicated in sepsis pathogenesis, i.e., systemic inflammation and cytokine release (Fig. 2A), endothelial cell activation and function (Fig. 2B), and coagulation activation (Fig. 2C). Concerning the “systemic inflammation” domain, plasma levels of tissue inhibitor of metalloproteinase-1, interleukin (IL)-6, IL-8 and IL-10 did not differ between age groups. Plasma CRP and matrix metalloproteinase (MMP)-8 concentrations showed an overall difference between age groups (*P* < 0.05) with the lowest levels in patients < 50 years. Considering that biomarker levels are influenced by the severity of acute disease [31], we performed an additional analysis adjusting for baseline disease severity; in this analysis, CRP and IL-10 were significantly different among age groups (*p* < 0.05) in which CRP was lowest in patients < 50 years and IL-10 showed an age-dependent decrease (Additional file 1: Table S4).

Concerning endothelial cell activation and function, the plasma levels of soluble E-selectin, soluble intracellular adhesion molecule-1 (ICAM-1), fractalkine (all reflecting endothelial cell activation [27]), angiopoietin-1, and the angiopoietin-2/1 ratio (both reflecting barrier function [27]) showed overall differences between age groups. Patients < 50 years demonstrated the most substantial deviations, except for soluble ICAM-1 and the angiopoietin-2/1 ratio (more substantial deviation in patients ≥ 50–< 60 years) (Fig. 2B). All significant differences in endothelial cell activation and function were robust to correction for baseline disease severity (Additional file 1: Table S4).

Concerning parameters of coagulation activation (D-dimer, prothrombin time, and the anticoagulant proteins antithrombin and protein C), no differences between age groups were observed. However, platelet counts showed an age-group-dependent increase, which was maintained in the disease severity adjusted model (Additional file 1: Table S4).

To determine the robustness of the results, we performed a sensitivity analysis using age as a continuous variable (Additional file 1: Table S5). The results of this approach largely reproduced the analysis in age decades except for CRP, MMP-8, and the angiopoietin-2/1 ratio which were non-significant in the sensitivity analysis (*p* = 0.08, *p* = 0.37 and *p* = 0.17 respectively).

### Blood leukocyte transcriptomes

**Fig. 3:**
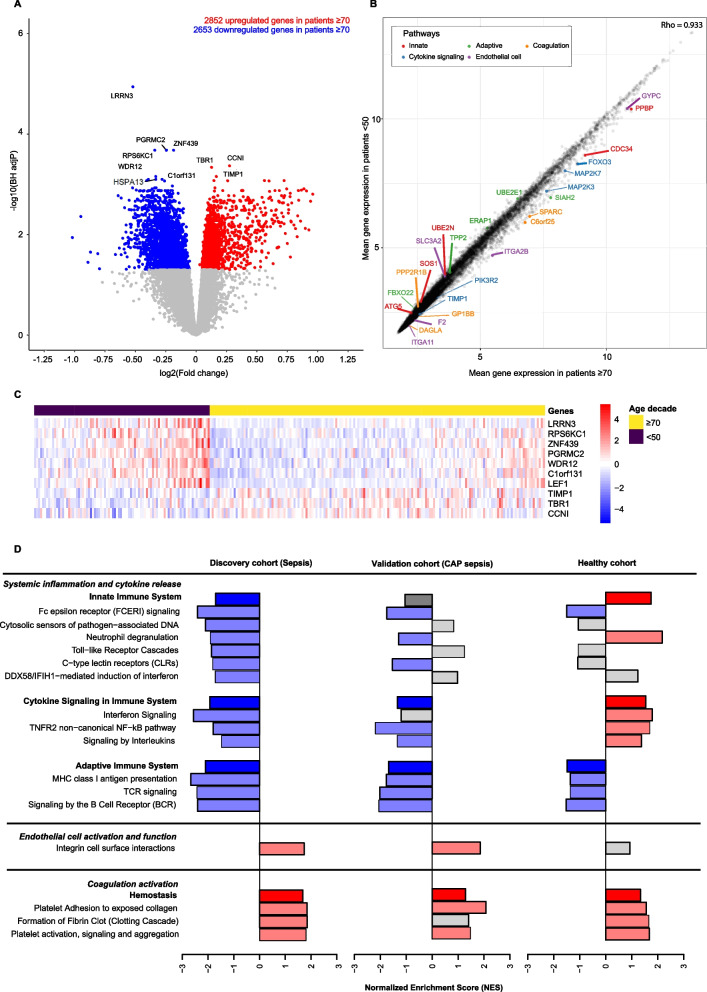
Gene expression and pathway analysis of sepsis patients ≥ 70 (*n* = 168) versus sepsis patients < 50 years of age (*n* = 88) with validation in and comparison to two independent cohorts (validation sepsis CAP cohort and healthy cohort). **A** Volcano plot portraying 5505 differentially expressed genes; 2852 higher and 2653 lower expressed genes in sepsis patients ≥ 70 years compared to sepsis patients < 50 years. The top 10 differentially expressed genes are labelled. **B** For each available gene, the mean gene expression in sepsis patients ≥ 70 years was correlated to the mean gene expression of that gene in patients < 50 years. The overall mean gene expression in sepsis patients ≥ 70 years was strongly correlated to the overall mean gene expression of sepsis patients < 50 years (Rho = 0.993). For every Reactome host response pathway of interest, the top 5 differentially expressed unique genes per pathway were colored. **C** Heat map of top 10 significantly differentially expressed genes in sepsis patients ≥ 70 compared to sepsis patients < 50 years. **D** Reactome Pathway analysis of all three cohorts (Discovery MARS sepsis cohort, validation sepsis CAP cohort, healthy cohort). The magnitude of expression is portrayed using Normalized Enrichment Scores. Red bars represent a significantly higher overall expression of that pathway in individuals ≥ 70 years compared to < 50 years. Blue bars represent a significantly lower overall expression of that pathway in individuals ≥ 70 years compared to < 50 years. Grey bars represent non-significantly different expressed pathways. The parent pathways are portrayed with increased density and followed by the contributing children pathways. The Reactome pathway: “Class I MHC mediated antigen processing & presentation” was abbreviated to “MHC class I antigen presentation”. The Reactome pathway “DDX58/IFIH1-mediated induction of interferon-alpha/beta” was abbreviated to “DDX58/IFIH1-mediated induction of interferon”

We compared the blood leukocyte transcriptomes of sepsis patients < 50 years (*n* = 88) to sepsis patients ≥ 70 years (n = 168). This analysis comprised the subgroup of sepsis patients enrolled during the first 1.5 years of this study with an infection likelihood of probable or definite (Additional file 1: Tables S6 and S7). Differential gene expression analysis revealed 5505 differentially expressed genes (DEGs) between patients < 50 and ≥ 70 years (Fig. 3A). The top 10 significantly DEGs are displayed in a heat map (Fig. 3C). The overall mean gene expression in sepsis patients ≥ 70 years was strongly correlated to the overall mean gene expression of sepsis patients < 50 years (Rho = 0.993; Fig. 3B). Sepsis patients ≥ 70 years demonstrated decreased expression of pathways related to “systemic inflammation and cytokine release” as compared to patients < 50 years, including crucial innate immunity pathways (e.g., Toll-like receptor cascades, C-type lectin receptors), cytokine signaling pathways (e.g., both interleukin and interferon signaling) and adaptive immunity pathways (e.g., B-cell and T-cell receptor signaling) (Fig. 3D, left panel). By contrast, sepsis patients ≥ 70 years appeared to have an increased expression of genes involved in endothelial cell activation and function (defined by the Reactome pathway “integrin cell surface interactions”), and “coagulation activation” (defined as hemostasis-related pathways in Reactome) compared to the sepsis patients < 50 years.

Next, we sought to validate these blood leukocyte transcriptome data in an independent patient cohort. To this end, we used a publicly available blood gene expression data set from critically ill patients with sepsis due to CAP, entailing 74 patients < 50 years and 167 patients ≥ 70 [30]. Pathway analyses in this independent cohort largely confirmed the data obtained in our patient cohort, with patients ≥ 70 years showing decreased expression of pathways related to cytokine signaling and the adaptive immune system, and increased expression of pathways related to endothelial cell activation and coagulation activation (Fig. 3D, middle panel). The only notable difference between both cohorts involved the innate immunity pathway, which was less down-regulated in patients ≥ 70 years in the CAP sepsis cohort.

In order to assess whether these differences in gene expression profiles are sepsis-driven or present in healthy subjects as a function of age, we analysed a publicly available data set of the blood transcriptomes of 77 healthy subjects < 50 years and 113 subjects ≥ 70 years [29]. Interestingly, healthy subjects ≥ 70 years displayed increased rather than decreased expression of pathways related to innate immunity and cytokine signaling (Fig. 3D, right panel), suggesting that the diminished expression of these pathways was sepsis-induced. Likewise, the increased expression of pathways related to endothelial cell activation and function detected in patients ≥ 70 was absent in healthy subjects ≥ 70 years. In contrast, the reduced expression of pathways related to adaptive immunity and the enhanced expression of pathways related to coagulation activation found in patients ≥ 70 was already present in healthy subjects ≥ 70 years.

### Weighted gene co-expression analysis

**Fig. 4:**
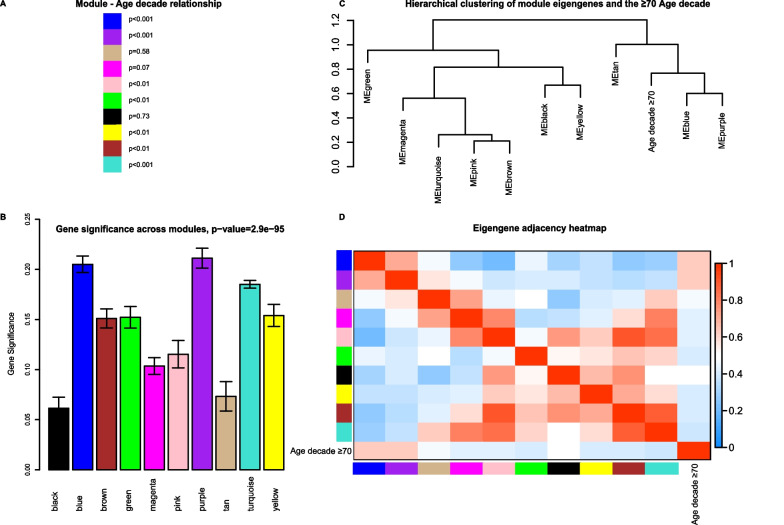
Module-module and module-trait associations. **A** Results of the Wilcoxson rank-test comparing the Module Eigengene (ME), defined as the first principal component of that module, between patients < 50 and ≥ 70 years. *P* values were multiple testing corrected using the Benjamini–Hochberg procedure. **B** Barplot of mean gene significance across modules. Higher gene significance in a module resembles a more substantial relationship of the module with a change in age decade. **C** Hierarchical clustering of module eigengenes and the ≥ 70 age decade. The cluster tree shows that the ≥ 70 age decade is related to the blue and the purple cluster. **D** Heatmap of the adjacencies in the eigengene network showing the module-module and module-trait (age decade ≥ 70) relationships. Modules are labelled by their corresponding colour. Red colours indicate high adjacency (positive correlation), blue colours indicate low adjacency (negative correlation)

**Fig. 5:**
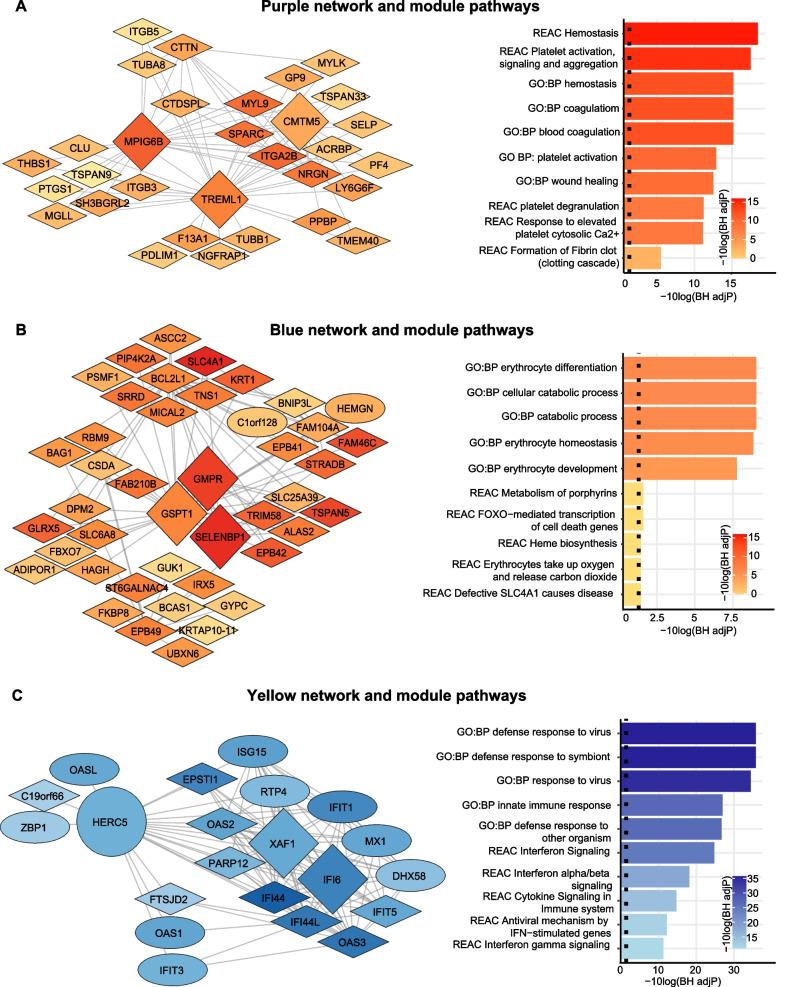
Gene co-expression networks of nodes with an edge of at least 0.2 of the purple, blue and yellow module, including the top 5 significantly different expressed pathways per pathway database. Gene co-expression networks visualised using Cytoscape 3.0 software. Networks, for which the edge threshold was set to 0.3, are displayed in the left panel. The node colours resemble the log2(Fold change) when comparing patients ≥ 70 to patients < 50 years. Blue colours indicate lower expression in patients ≥ 70, red colours indicate higher expression in patients ≥ 70. The diamond shape resembles a Benjamini–Hochberg (BH)-corrected p-value < 0.05, and ellipses resemble non-significantly different expressed genes. The top 3 hub genes are represented by an increase in size. The right panel portrays the top 5 pathways within the Gene Ontology Biological Process (GO:BP) and top 5 pathways within the Reactome database (REAC) of all genes within the module (independent of the edge). **A** Purple network and module pathway analysis. **B** Blue network and module pathway analysis for which the edge threshold of the network was set to 0.33 due to the gene size of the module. **C** Yellow network and module pathway analysis

At last, we performed a weighted gene co-expression analysis in which we incorporated all whole blood transcriptome data of the MARS cohort (Additional file 2: Fig. S1). No outliers were detected (Additional file 2: Fig. S2). The optimal power (*β*) for a scale-free network was estimated to be 13 (Additional file 2: Fig. S3). Twelve modules of co-expressed genes were identified, of which ten remained after merging similar modules (Additional file 2: Fig. S4). After the removal of genes with a low Module Membership, modules varied from 408 genes (turquoise module) to 26 genes (tan module) (see Additional file 3 for complete annotation). Seven of the ten modules were significantly different between patients < 50 and ≥ 70 years. Two modules were positively correlated with an increase in age group (purple and blue) and five modules inversely correlated with an increase in age group (turquoise, yellow, green, brown, pink) (Fig. 4). Functional enrichment analysis demonstrated that the purple module consisted of genes involved in hemostasis and platelet activation, the blue module of genes involved in catabolic processes, erythrocytes, and heme biosynthesis, the yellow module of genes involved in interferon and cytokine signaling, the turquoise module of genes involved in metabolism and transport, the green module of genes involved in T-cell and lymphocyte activation, the brown module of genes involved in RNA/DNA metabolism, and the pink module of genes involved in autophagy and stress (see Additional file 3 for complete annotation). Based on gene significance and functional enrichment results (Figs. 4A + B and 5C respectively), the purple (hemostasis and platelet activation), blue (catabolic processes, erythrocytes, and heme biosynthesis) and yellow (interferon and cytokine signaling) modules were deemed most relevant. A network view of these modules, including hub genes and significantly different pathways, is depicted in Fig. 5. The significantly different pathways of the other modules are depicted in Fig. S5 (Additional file 2).

## Discussion

Although it is well established that older age is associated with increased sepsis mortality, investigations on the influence of age on host response aberrations are largely limited to plasma cytokines. We here report a combined analysis of 16 host response biomarkers indicative of alterations in key pathophysiological domains of sepsis and blood leukocyte transcriptome profiles in a large prospectively enrolled cohort of sepsis patients stratified according to age decades. While we confirm the findings of earlier studies that demonstrate that systemic inflammation and cytokine release during sepsis are not or hardly affected by age [18–24], we provide novel evidence that older age is associated with relatively mitigated endothelial cell activation and a less disturbed endothelial barrier function. Moreover, blood transcriptome analysis revealed age-dependent differences that were partially sepsis-driven (reduced expression of pathways relating to cytokine signaling and increased expression of pathways relating to integrin cell surface interactions) and partially already present in healthy elderly persons (reduced expression of pathways relating to adaptive immunity, and increased expression of pathways implicated in hemostasis). Our study represents the most comprehensive analysis on the influence of age on the host response during sepsis to date, and argues for a broader implementation of ageing cells and organisms in future investigations on pathophysiological mechanisms at play in sepsis.

In line with previous research [3, 5], older age was a risk factor for mortality, independent of age-related comorbidities and disease severity. We did not find a strong association between ageing and markers of systemic inflammation and cytokine release, which is in accordance with previous investigations showing little or no association between age and inflammation markers in the context of sepsis and CAP [18–24]. One study reported higher plasma levels of two cytokine antagonists (IL-1 receptor antagonist, soluble tumor necrosis factor receptor type 1) in older patients with sepsis [17]. Considering that the plasma concentrations of inflammatory biomarkers positively correlate with the severity of disease [31], one might argue that older patients (demonstrating higher SOFA scores and a higher proportion of shock upon presentation) showed a relatively blunted systemic inflammatory and cytokine response. Similarly, our group previously reported similar cytokine levels in older patients hospitalized for CAP in spite of markedly higher pneumonia severity indexes [21]. However, adjusting for disease severity did not expose such an age-dependent association, with the exception of the anti-inflammatory cytokine IL-10, which showed an age-dependent decrease in the model adjusting for baseline disease severity. Collectively, these data suggest that ageing does not have a strong effect on systemic inflammatory and cytokine responses in sepsis.

Thus far, knowledge of endothelial cell and coagulation activation in patients with sepsis in the context of ageing was highly limited. Animal models have suggested an association between ageing and endothelial responses. For instance in experimental endotoxemia and sepsis, aged mice showed increased plasma concentrations of some endothelial adhesion molecules (e.g., E-selectin) [40, 41], while others (e.g., sICAM) are similar [40]. Moreover, aged mice demonstrated increased fibrin formation, similar D-dimer and lower Protein C concentrations in sepsis models [42, 43]. These results are difficult to translate to humans, however, considering species differences [44, 45]. For example, while in human sepsis no association of ageing with cytokines is present [18–24], such an association does exist in mice [46].

We show that in sepsis patients, ageing was associated with a striking decrease of all measured endothelial cell activation markers (soluble E-selectin, soluble ICAM-1, fractalkine), an increase in angiopoietin-1 concentrations and a decrease of the angiopoietin-2/1 ratio. These results suggest that older sepsis patients may exhibit attenuated endothelial cell activation and barrier function disturbances during sepsis, despite a higher disease severity (which is expected to aggravate this aberrations). These dampened endothelial responses in older patients with sepsis are remarkable considering that ageing per se is associated with vascular dysfunction due to multiple underlying mechanisms, including oxidative stress and chronic low-grade inflammation, and characterized by sustained activation of the master regulator of inflammation nuclear factor (NF)-ĸB, increased expression of inflammatory cytokines and adhesion molecules, and enhanced proinflammatory endothelial cell-leukocyte interactions [47, 48]. Possibly, this seeming paradox can be explained by a phenomenon that has been named endothelial tolerance in analogy to the well-described tolerance of monocytes and macrophages [25, 49]: i.e., re-challenging endothelial cells with lipopolysaccharide in vitro was associated with a reduced responsiveness, characterized by decreased accumulation of NF-κB, leukocyte adhesion, and E-selectin expression [50, 51].

We did not detect differences between age groups in any of the coagulation markers, which is in contrast with one study in patients with CAP [22]. Conceivably, the modest age-related differences in coagulation activation observed in CAP may be obscured by the more extensive coagulation abnormalities present in sepsis [22, 26, 52]. We did observe higher platelet counts in older septic patients which persisted after correcting for disease severity. Of note, platelet counts mostly were in physiological range, and lower, rather than higher, platelet numbers have been linked to worse clinical outcomes and alterations in the host response during sepsis [27, 53, 54].

Analysis of the blood leukocyte transcriptome of sepsis patients revealed that ageing was associated with decreased expression of genes involved in innate immunity, cytokine signaling and adaptive immunity, and increased expression of genes involved in endothelial cell activation and coagulation activation. Except for the innate immunity pathway, these associations were validated in an independent sepsis CAP cohort [30]. Given these results, particularly anti-inflammatory agents may not be of benefit to older sepsis patients, especially if the patients selection is based on disease severity scores. By contrast, within healthy individuals, ageing was associated with enhanced expression of innate immunity and cytokine signaling pathways, possibly reflecting inflammageing [14–16]. Therefore, the diminished expression of these pathways in sepsis patients may be sepsis-induced. Notably, the diminished expression of cytokine signalling pathways in blood transcriptomes in older sepsis patients was detected, while plasma cytokines were mostly not altered in this age group, suggesting that blood leukocytes are less responsive to cytokines in this age group. Of interest, the leukocyte transcriptomes of older sepsis patients displayed increased expression of the “integrin cell surface interactions” pathway, which was not present in older healthy subjects. Together these data suggest that certain aspects of leukocyte functions are diminished in older sepsis patients.

A weighted gene-co expression analysis of the blood leukocyte transcriptome confirmed our results. In this unsupervised analysis, we equally observed a positive association of ageing with the expression of genes involved in hemostasis and a negative association of ageing with the expression of genes involved in innate and adaptive immunity and cytokine and interferon signaling. Within this analysis, we also observed an association of ageing with increased expression of genes involved in catabolic processes, erythrocytes, and heme biosynthesis. Recent papers suggest heme may play a central role in the pathogenesis of sepsis, stressing the relevance of this finding [55]. In fact, plasma heme has been shown to increase tissue damage in sepsis, promote microbial growth, and sensitize cells to programmed cell death under proinflammatory conditions [55–57].

Our study has strengths and limitations. First, our blood leukocyte transcriptome analysis was validated in an independent CAP sepsis cohort and compared with the transcriptome of healthy individuals. Second, data is derived from a large prospectively enrolled ICU sepsis population according to strict criteria with highly detailed epidemiologic data and few missing data points. Considering that all patients with suspected infection were included using an opt-out method, we believe that our cohort truly reflects the sepsis population at that moment in time. Notably, inclusion of patients was done in two tertiary ICUs in the Netherlands, which may impact generalizability. Certain aspects of sepsis management may have changed over time as the data was collected several years ago (2011–2014) [58]. Given that our data was derived from plasma and whole blood transcriptomes, our results do not fully reflect the complexity of the host response in sepsis, particularly relating to inflammatory, endothelial, and procoagulant changes occurring outside the blood compartment.

Our results show that the host response to sepsis is affected by age. Despite the higher disease severity among older sepsis patients, these patients (relative to younger patients) had similar plasma concentrations of inflammation, cytokine and coagulation markers, and evidence for decreased endothelial activation and a less disturbed endothelial barrier function. Additionally, the blood transcriptome of older patients demonstrated a sepsis-induced decreased expression of cytokine signaling pathways and a higher expression of gene pathways related to hemostasis. Precision medicine, representing diagnostic and therapeutic strategies that take specific patient features into consideration, has not been widely adopted in the field of sepsis [59]. Key in precision medicine is predictive enrichment, which indicates selection of patients who are more likely to react positively to a particular therapy based on a biological mechanism [59]. This study suggests that age is an important factor to consider in future sepsis trials that target the immune and/or endothelial cell response.

## Supplementary Information

**Additional file 1: Table S1.** Summary of infections categorized as “Other infection site” or “Unknown infection site”. **Table S2.** Baseline characteristics of critically ill sepsis patients stratified by age decades (host response biomarker cohort)**. Table S3**. Clinical outcomes of critically ill sepsis patients stratified by age decades (host response biomarker cohort). **Table S4**. Association of ageing with biomarker concentrations after correcting for baseline disease severity. **Table S5**. Sensitivity analysis of biomarkers of critically ill sepsis patients using a linear regression model and age on a continuous scale. **Table S6**. Baseline characteristics of critically ill sepsis patients (whole blood transcriptome cohort). **Table S7.** Clinical outcomes of critically ill sepsis patients (whole blood transcriptome cohort).

**Additional file 2: Fig. S1**. Flowchart MARS cohort. **Fig. S2.** Hierarchical average linkage clustering to detect outlier samples. **Fig. S3**. Weighted gene co-expression network analysis (WGCNA) network construction. **Fig. S4**. Weighted gene co-expression network analysis cluster dendogram and cluster assignment. **Fig. S5**. Top 5 significantly different expressed pathways per pathway database of the turquoise, green, pink and brown modules.

**Additional file 3: Sheet 1**. Gene annotation per module. **Sheet 2**. Purple module pathway analysis, **Sheet 3**. Blue module pathway analysis. **Sheet 4.** Yellow module pathway analysis. **Sheet 5.** Turquoise module pathway analysis. **Sheet 6**. Green module pathway analysis. **Sheet 7**. Brown module pathway analysis. **Sheet 8**. Pink module pathway analysis.

## Data Availability

Both the data of our cohort (MARS) and the cohort of healthy individuals are available at the Gene Expression Omnibus public repository of NCBI under accession number GSE65682 and GSE33828 respectively [29]. The CAP sepsis cohort is available at ArrayExpress under accession numbers E-MTAB-4421 and E-MTAB-4451 [30].

## Abbreviations

ANCOVA: Analysis of covariance
ANOVA: Analysis of variance
APACHE IV APS: Acute physiology and chronic health evaluation IV acute physiology
ARDS: Acute respiratory distress syndrome
BH adjustment: Benjamini–Hochberg adjustment
BMI: Body mass index
CAP: Community-acquired pneumonia
CRP: C-reactive protein
DEG: Differentially expressed genes
ICU: Intensive care unit
IL-6: Interleukin-6
IL-8: Interleukin-8
IL-10: Interleukin-10
MMP-8: Matrix metalloproteinase-8
NF-ĸB: Nuclear factor kappa-light-chain-enhancer of activated B cells
PT: Prothrombin time
SOFA: Sequential organ failure
sICAM-1: Soluble intercellular adhesion molecule-1
TIMP-1: Tissue inhibitor of metalloproteinase-1
WGCNA: Weighted gene co-expression network analysis
