# Supplementary material for: Association between age and the host response in critically ill patients with sepsis
Source: Crit Care. 2022 Dec 13;26:385. doi: 10.1186/s13054-022-04266-9 (PMC9747080; doi:10.1186/s13054-022-04266-9)
Supplement: Supplementary file 1 — Additional file 1: Table S1. Summary of infections categorized as “Other infection site” or “Unknown infection site”. Table S2. Baseline characteristics of critically ill sepsis patients stratified by age decades (host response biomarker cohort). Table S3. Clinical outcomes of critically ill sepsis patients stratified by age decades (host response biomarker cohort). Table S4. Association of ageing with biomarker concentrations after correcting for baseline disease severity. Table S5. Sensitivity analysis of biomarkers of critically ill sepsis patients using a linear regression model and age on a continuous scale. Table S6. Baseline characteristics of critically ill sepsis patients (whole blood transcriptome cohort). Table S7. Clinical outcomes of critically ill sepsis patients (whole blood transcriptome cohort). [file 13054_2022_4266_MOESM1_ESM.docx]

# **Additional file 1**

# **Supplementary methods and data**

# **Association between age and the host response in critically ill patients with sepsis**

Authors
Erik H.A. Michels, Joe M. Butler, Tom D.Y. Reijnders, Olaf L. Cremer, Brendon P. Scicluna, Fabrice Uhel, Hessel Peters-Sengers, Marcus J. Schultz, Julian C. Knight, Lonneke A. van Vught, Tom van der Poll, on behalf of the MARS consortium

# **Table of contents**

[**Table of contents** 2](#_Toc115709529)

[**List of tables in this Additional file 1** 3](#_Toc115709530)

[**List of tables in Additional file 2 (excel file)** 3](#_Toc115709531)

[1. Additional methods 4](#_Toc115709532)

[1.1 Definitions of comorbidities and outcomes 4](#_Toc115709533)

[1.2 Assays 4](#_Toc115709534)

[1.3 Whole Blood Gene Expression Microarrays 5](#_Toc115709535)

[1.4 Detailed information on statistical and bioinformatics analysis 5](#_Toc115709536)

[1.5 Exact Reactome pathway annotations 8](#_Toc115709537)

[1.6 Availability of gene expression cohorts 8](#_Toc115709538)

[2. Summary of infections categorized as “Other infection site” or “Unknown infection site” 9](#_Toc115709539)

[3. Demographics and clinical outcomes of host response biomarker cohort 10](#_Toc115709540)

[4. Association of ageing with biomarker concentrations after correcting for baseline disease severity 12](#_Toc115709541)

[5. Sensitivity analysis 13](#_Toc115709542)

[6. Demographics and clinical outcomes of whole blood transcriptome cohort 14](#_Toc115709543)

[7. References 16](#_Toc115709544)

# **List of tables in this Additional File 1**

Supplementary Table S1: Summary of infections categorized as “Other infection site” or “Unknown infection site”..........................................................................................................9
Supplementary Table S2: Baseline characteristics of critically ill sepsis patients stratified
by age decades (host response biomarker cohort)10

Supplementary Table S3: Clinical outcomes of critically ill sepsis patients stratified by
age decades (host response biomarker cohort) 11
Supplementary Table S4: Association of ageing with biomarker concentrations after
correcting for baseline disease severity ..................................................................................12
Supplementary Table S5: Sensitivity analysis of biomarkers of critically ill sepsis
patients using a linear regression model and age on a continuous scale13
Supplementary Table S6: Baseline characteristics of critically ill sepsis patients (whole
blood transcriptome cohort)14
Supplementary Table S7: Clinical outcomes of critically ill sepsis patients (whole
blood transcriptome cohort)15

# **List of tables in Additional file 3 (excel file)**

Additional file 2 sheet 1: Gene annotation per module
Additional file 2 sheet 2: Purple module pathway analysis
Additional file 2 sheet 3: Blue module pathway analysis
Additional file 2 sheet 4: Yellow module pathway analysis
Additional file 2 sheet 5: Turquoise module pathway analysis
Additional file 2 sheet 6: Green module pathway analysis
Additional file 2 sheet 7: Brown module pathway analysis
Additional file 2 sheet 8: Pink module pathway analysis

## Additional methods

### Definitions of comorbidities and outcomes

Cardiovascular insufficiency was defined as a medical history of congestive heart failure, chronic cardiovascular disease, peripheral vascular disease, or cerebrovascular disease. Malignancy was defined as a medical history of either a non-metastatic solid tumor, metastatic malignancy, or hematologic malignancy. Immunocompromised was defined as a medical history of either an immune deficiency, AIDS, HIV positivity, chronic use of corticosteroids, or recent chemotherapy. Renal insufficiency was defined as a history of chronic renal insufficiency, chronic intermitted hemodialysis, or continuous ambulatory peritoneal dialysis. The Charlson comorbidity index was determined without the use of age [1]. Septic shock was defined as the use of >0.1µg/kg/min norepinephrine during at least 50% of the ICU day due to hypotension. The causative pathogen was determined based on all available microbiology results. Acute kidney injury and acute respiratory distress syndrome were defined using strict pre-set criteria [2,3]. Complications that started two days or more after ICU admission were defined as ICU-acquired. Length of hospital stay was calculated starting from ICU admission.

### 1.2 Assays

All biomarker measurements were done in EDTA anti-coagulated plasma obtained within 16 hours after ICU admission. Biomarker measurements were limited to sepsis patients with an infection likelihood of definite or probable, defined as described in detail previously [4], enrolled during the first 2.5 years of the MARS project. Interleukin-6, interleukin-8, interleukin-10, soluble Intercellular adhesion molecule-1, soluble E-selectin, and fractalkine were measured by FlexSet cytometric bead array (BD Biosciences, San Jose, CA) using FACS Calibur (Becton Dickenson, Franklin Lakes, NJ). Protein C, antithrombin, Matrix metalloproteinase-8, Tissue inhibitor of metalloproteinase-1, Angiopoietin-1, Angiopoietin-2 (all R&D systems, Abingdon UK), and D-dimer (Procartaplex, eBioscence, San Diego, CA) were measured by Luminex multiplex assay using BioPlex 200 (BioRad, Hercules, CA). C-reactive protein was determined by immunoturbidimetric assay (Roche diagnostics), Prothrombin time by using a photometric method with Dade Innovin Reagent (Siemens Healthcare Diagnostics). Normal values were obtained from 27 sex-matched healthy volunteers, of whom written informed consent was obtained, except for CRP, platelet count, and prothrombin time, for which routine laboratory reference values were used.

### 1.3 Whole Blood Gene Expression Microarrays

Within 24 hours after admission, whole blood was collected in PAXgene tubes (Becton-Dickinson, Breda, the Netherlands) and stored at -80°C. RNA analysis was limited to sepsis patients with an infection likelihood of definite or probable enrolled during the first 1.5 years of the MARS project [4]. Total RNA was isolated using the PAXgene blood mRNA kit (Qiagen, Venlo, the Netherlands). RNA, with an integrity number of at least 6, was processed and hybridized to the Affymetrix Human Genome U219 96-array and scanned using the GeneTitan instruments at the Cologne Center for Genomics (CCG), Cologne, Germany, as described in detail [5].

### 1.4 Detailed information on statistical and bioinformatics analysis

Data distributions were assessed by histograms and quantile-quantile plots (Q-Q plots). Categorical variables were analysed using a Chi-square test of independence, non-normal continuous data using a Kruskal-Wallis test, and normally distributed continuous using an analysis of variance (ANOVA). For post hoc testing of non-normal data, a Dunn’s test of multiple comparisons using rank sums; for post hoc testing of normally distributed continuous data, a Tukey post hoc test was conducted. Differences in 30-day survival were visualized by Kaplan-Meier curves and assessed using both an unadjusted and adjusted Cox proportional-hazards model. The proportionality of hazards was assessed using Schoenfeld residuals and linearity of covariates by inspecting martingale residuals.

Biomarker and routine laboratory data were normalized using a box-cox transformation. Prior research has shown that ageing is associated with an increase in sepsis severity [6]. Additionally, the release of host response biomarkers in sepsis is often proportional to the severity of disease [7]. Therefore, we performed a secondary analysis in which we sought to evaluate if ageing associated host response aberrations changes when correcting the ageing-driven higher disease severity. The severity adjusted ANCOVA included the Sequential Organ Failure Score (SOFA) score, Acute Physiology and Chronic Health Evaluation IV Acute Physiology (APACHE IV APS) score, and prevalence of shock and acute kidney injury on ICU admission. For the ANCOVA, homogeneity of the regression slopes was evaluated by analyzing interaction terms of age and each covariate followed by visual inspection of scatter plots with regression lines. The normality of the residuals was assessed by visual inspection of histograms and quantile-quantile plots. At last, homogeneity of the residual variances was tested using the Levene’s test, while multicollinearity was assessed using variance inflation factors with a cutoff of 2.5. When analyzing overall differences in platelet count among age groups in the disease severity model, the SOFA score was replaced by a modified SOFA score in which the coagulation element was excluded.

For the sensitivity analysis, age was modelled as a continuous variable. Linearity of age with biomarker concentrations was assessed using a restricted cubic spline regression analysis (with n=3 knots at default location). Nonlinearity was assessed by a Wald test of the nonlinear terms of the restricted cubic spline (p<0.05 indicating nonlinearity). Biomarkers that showed a linear relationship with age were analysed using a linear regression analysis, nonlinear biomarkers were analysed using a restricted cubic spline function with 3 inner knots. All biomarker analyses (ANOVA, ANCOVA, regression analysis) were multiple testing corrected using the Benjamini-Hochberg (BH) method for the conduction of 17 tests (testing of 17 biomarkers).

A weighted gene co-expression network analysis (WGCNA) was performed to find clusters of highly correlating genes (often termed modules) across all transcriptomic samples of the MARS cohort. The rationale and a step-by-step approach to an adequate WGCNA are described in detail by Langfelder and Horvarth (2008) [8]. We removed low expressed genes by selecting the 10% most variable genes [9]. Outlier samples were detected using hierarchical average linkage clustering. After removing outliers and lowly expressed genes, we aimed to construct a gene co-expression network. First, we constructed a scale-free network. The appropriate power value to obtain a scale-free network was based on the “pickSoftThreshold” function in which the network type was set to signed. The scale independence and mean connectivity was tested using a power value ranging from 1 to 20 [8]. Using the obtained power value, the Pearson’s correlation matrix of all gene pairs was transformed into an adjacency matrix and topological overlap matrix. Modules, which represent genes with high topological overlap, were identified using the dynamic tree cut algorithm with a minimal cluster size of 40 genes [8]. Similar modules were merged based on a correlation coefficient greater than 0.75 [10]. Next, eigengene-based connectivity (KME), a continuous measure of Module Membership [11], was calculated for each gene per module. To clarify, the KME/MM reflects the correlation of a gene’s expression profile with the first principal component of a module [8]. Genes that are strongly assigned to a module will demonstrate a strong correlation (high KME/MM) with the first principal component of that module, while genes that do not belong to that module will show a weak correlation (low KME/MM). For the current analysis, genes weakly correlated to all obtained modules (|KME|<0.7) were not assigned [12]. Relevant modules were identified based on two methods. First, the first principal component of that module [8], often termed module eigengene (ME), was compared between patients <50 and ≥70 using a Wilcoxon rank-sum test. Second, the average absolute gene significance of all genes in a given module was evaluated. Modules significant in both methods were further explored. Functional profiling was conducted in g:Profiler using on KME based ordered query [13]. Due to the ordering of the query on KME, genes strongly assigned to a module have more weight in the functional enrichment analysis. A BH adjusted p-value <0.05 of a pathway within the Gene Ontology Biological Process or Reactome database was considered significant [14,15]. At last, the Maximal Clique Centrality algorithm of the Cytospace plugin CytoHubba was used to identify hub genes [16].

### 1.5 Exact Reactome pathway annotations

Pathway analysis was performed using the Reactome database [15]. First, to reflect inflammation and cytokine release, we analysed pathways within the Innate Immune System (R-HSA-168249), Adaptive Immune System (R-HSA-1280218), and Cytokine Signaling in Immune system (R-HSA-1280215) pathways. Second, to reflect endothelial cell activation and function, we analysed the Cell surface Interaction at the Vascular wall (R-HSA-202733) and Integrin cell surface interactions (R-HSA-216083) pathways. Last, to reflect coagulation activation, we analysed the pathways within the Hemostasis pathway (R-HSA-109582) [15].

### 1.6 Availability of gene expression cohorts

Both our cohort (MARS) and the cohort of healthy individuals are available at the Gene Expression Omnibus public repository of NCBI under accession number GSE65682 and GSE33828 respectively [17]. The CAP sepsis cohort is available at ArrayExpress under accession numbers E-MTAB-4421 and E-MTAB-4451 [9]

## Summary of infections categorized as “Other infection site” or “Unknown infection site”

Supplementary Table S1 Summary of infections categorized as “Other infection site” or “Unknown infection site”

|  | **<50 years** | **≥50 - <60 years** | **≥60 - <70 years** | **≥70 years** |
| --- | --- | --- | --- | --- |
| **n** | 421 | 368 | 545 | 618 |
| **Other infection sites, n (%)** |  |  |  |  |
| **Bones and joints** | 7 (1.7) | 4 (1.1) | 7 (1.3) | 9 (1.5) |
| **Reproductive tract** | 1 (0.2) | 0 (0.0) | 1 (0.2) | 0 (0.0) |
| **Mediastinum** | 2 (0.5) | 15 (4.1) | 22 (4.0) | 19 (3.1) |
| **Ear** | 0 (0.0) | 0 (0.0) | 1 (0.2) | 0 (0.0) |
| **Oral** | 7 (1.7) | 1 (0.3) | 4 (0.7) | 2 (0.3) |
| **Unknown infection sites, n (%)** |  |  |  |  |
| **Unknown** | 11 (2.6) | 8 (2.2) | 10 (1.8) | 19 (3.1) |
| **Systemic viral infection** | 1 (0.2) | 2 (0.5) | 2 (0.4) | 0 (0.0) |
| **Primary bacteremia** | 4 (1.0) | 7 (1.9) | 11 (2.0) | 12 (1.9) |

## Demographics and clinical outcomes of host response biomarker cohort

Supplementary Table S2: Baseline characteristics of critically ill sepsis patients stratified by age decades (host response biomarker cohort)

|  | **<50 years** | | **≥50 - <60 years** | **≥60 - <70 years** | **≥70 years** | **p-value** |
| --- | --- | --- | --- | --- | --- | --- |
| **n** | 187 | | 163 | 259 | 280 |  |
| **Demographics** |  | |  |  |  |  |
| **Age years, median [IQR]** | 40.00 [30.00, 47.00] | | 55.00 [53.00, 58.00] | 64.00 [62.00, 67.00] | 75.00 [72.00, 79.00] | <0.001 |
| **Male sex, n (%)** | 101 (54.0) | | 100 (61.3) | 165 (63.7) | 171 (61.1) | 0.213 |
| **White race, n (%)** | 143 (76.5) | | 146 (89.6) | 231 (89.9) | 264 (94.6) | <0.001 |
| **BMI, median [IQR]** | 23.53 [21.43, 26.23] | | 24.59 [21.31, 27.70] | 25.35 [22.84, 29.19] | 25.21 [23.01, 27.92] | <0.001 |
| **Medical admission, n (%)** | 135 (72.2) | | 117 (71.8) | 194 (74.9) | 204 (72.9) | 0.883 |
| **Comorbidity** |  | |  |  |  |  |
| **Charlson score *, median [IQR]** | 0.00 [0.00, 2.00] | | 2.00 [0.00, 3.00] | 2.00 [0.00, 3.00] | 2.00 [1.00, 3.00] | <0.001 |
| **Cardiovascular, n (%)** | 23 (12.3) | | 32 (19.6) | 65 (25.1) | 90 (32.1) | <0.001 |
| **Respiratory insufficiency, n (%)** | 10 (5.3) | | 9 (5.5) | 18 (6.9) | 23 (8.2) | 0.584 |
| **Hypertension, n (%)** | 23 (12.3) | | 38 (23.3) | 78 (30.1) | 125 (44.6) | <0.001 |
| **Diabetes, n (%)** | 18 (9.6) | | 26 (16.0) | 53 (20.5) | 74 (26.4) | <0.001 |
| **Malignancy, n (%)** | 38 (20.3) | | 51 (31.3) | 75 (29.0) | 76 (27.1) | 0.100 |
| **Renal disease, n (%)** | 15 (8.0) | | 28 (17.2) | 39 (15.1) | 44 (15.7) | 0.052 |
| **Immunocompromised, n (%)** | 64 (34.2) | | 54 (33.3) | 63 (24.3) | 45 (16.1) | <0.001 |
| **Chronic medication, n (%)** |  | |  |  |  |  |
| **Anticoagulants** | 16 (8.6) | | 20 (12.3) | 43 (16.6) | 56 (20.1) | 0.005 |
| **Antiplatelet drugs** | 13 (7.6) | | 24 (15.4) | 70 (28.9) | 107 (39.5) | <0.001 |
| **Site of infection, n (%)** |  | |  |  |  |  |
| **Cardiovascular** | 11 (5.9) | | 4 (2.5) | 11 (4.2) | 13 (4.6) | 0.474 |
| **Pulmonary** | 75 (40.1) | | 55 (33.7) | 98 (37.8) | 110 (39.3) | 0.614 |
| **Urinary** | 11 (5.9) | | 5 (3.1) | 19 (7.3) | 16 (5.7) | 0.337 |
| **Skin** | 11 (5.9) | | 12 (7.4) | 6 (2.3) | 9 (3.2) | 0.043 |
| **Abdominal** | 25 (13.4) | | 30 (18.4) | 44 (17.0) | 51 (18.2) | 0.518 |
| **Central nervous system** | 5 (2.7) | | 2 (1.2) | 9 (3.5) | 2 (0.7) | 0.107 |
| **Other infection site** † | 6 (3.2) | | 7 (4.3) | 16 (6.2) | 10 (3.6) | 0.385 |
| **Mixed infection** | 41 (21.9) | | 46 (28.2) | 51 (19.7) | 70 (25.0) | 0.193 |
| **Unknown site** ‡ | 9 (4.8) | | 8 (4.9) | 8 (3.1) | 6 (2.1) | 0.311 |
| **Causative pathogen primary site of infection** §**, n (%)** | |  |  |  |  |  |
| **Gram-positive bacteria** | 84 (44.9) | | 69 (42.3) | 111 (42.9) | 111 (39.6) | 0.714 |
| **Gram-negative bacteria** | 71 (38.0) | | 82 (50.3) | 120 (46.3) | 122 (43.6) | 0.116 |
| **Fungi** | 21 (11.2) | | 23 (14.1) | 38 (14.7) | 23 (8.2) | 0.094 |
| **Virus** | 19 (10.2) | | 13 (8.0) | 17 (6.6) | 9 (3.2) | 0.022 |
| **Other** | 14 (7.5) | | 4 (2.5) | 7 (2.7) | 10 (3.6) | 0.040 |
| **Unknown** | 25 (13.4) | | 21 (12.9) | 37 (14.3) | 60 (21.4) | 0.032 |
| **Disease severity on ICU admission** | | | | | | |
| **APACHE IV APS, median [IQR]** | 66.00 [49.00, 87.00] | | 73.00 [56.00, 94.00] | 67.00 [50.00, 86.50] | 71.00 [55.00, 87.00] | 0.046 |
| **SOFA score** ll**, median [IQR]** | 7.00 [4.00, 9.50] | | 8.00 [6.00, 10.00] | 7.00 [4.00, 9.00] | 7.00 [5.75, 9.00] | 0.066 |
| **Shock, n (%)** | 38 (20.3) | | 52 (31.9) | 62 (23.9) | 98 (35.0) | 0.001 |
| **ARDS, n (%)** | 54 (28.9) | | 50 (30.7) | 68 (26.3) | 87 (31.1) | 0.628 |
| **Acute kidney injury, n (%)** | 57 (30.5) | | 69 (42.3) | 91 (35.1) | 134 (47.9) | 0.001 |

Abbreviations: BMI: Body Mass Index, APACHE IV APS: Acute Physiology and Chronic Health Evaluation IV Acute Physiology score, SOFA: Sequential Organ Failure Score, ARDS: acute respiratory distress syndrome.
* The Charlson score was calculated without the age component
† Other infection sites consisted of infections of bones and joints, the reproductive tract, mediastinum, the ear, throat or mouth.
‡ Unknown site of infection consisted of infections of unknown source, systemic viral infections, and primary bacteremia.
§ Causative organisms of the primary site of infection do not add up to 100% as some patients suffered from multiple pathogens at the primary site.
ll The SOFA score was calculated without the Central Nervous System component

Supplementary Table S3: Clinical outcomes of critically ill sepsis patients stratified by age decades (host response biomarker cohort)

|  | **<50 years** | **≥50 - <60 years** | **≥60 - <70 years** | **≥70 years** | **p-value** |
| --- | --- | --- | --- | --- | --- |
| n | 187 | 163 | 259 | 280 |  |
| **ICU-acquired complications, n (%)** | | | | |  |
| **Shock** | 29 (15.5) | 26 (16.0) | 55 (21.2) | 44 (15.7) | 0.270 |
| **ARDS** | 9 (4.8) | 10 (6.1) | 11 (4.2) | 14 (5.0) | 0.857 |
| **Acute kidney injury** | 14 (7.5) | 10 (6.1) | 35 (13.5) | 19 (6.8) | 0.015 |
| **ICU-acquired infections** | 20 (10.7) | 21 (12.9) | 28 (10.8) | 33 (11.8) | 0.903 |
| **Length of stay, median [IQR]** | | | | | |
| **ICU stay, days *** | 4.19 [1.73, 10.50] | 6.05 [2.82, 10.97] | 4.85 [2.06, 9.79] | 4.82 [2.25, 9.51] | 0.221 |
| **Hospital stay, days †** | 24.35 [9.75, 45.52] | 26.30 [13.39, 43.37] | 22.14 [13.53, 47.21] | 20.27 [12.06, 35.96] | 0.419 |
| **Mortality, n (%)** |  |  |  |  |  |
| **ICU** | 27 (14.4) | 39 (23.9) | 45 (17.4) | 73 (26.1) | 0.007 |
| **Hospital** | 40 (21.4) | 56 (34.4) | 77 (29.8) | 113 (40.4) | <0.001 |
| **Day 30** | 36 (19.3) | 51 (31.3) | 58 (22.5) | 109 (38.9) | <0.001 |
| **Day 60** | 49 (26.2) | 57 (35.0) | 78 (30.2) | 127 (45.4) | <0.001 |
| **Day 90** | 55 (29.4) | 65 (39.9) | 88 (34.1) | 137 (48.9) | <0.001 |
| Abbreviations: ARDS: acute respiratory distress syndrome, ICU: intensive care. * Length of ICU stay was only calculated in those who survived the entire ICU admission  † Length of hospital was only calculated in those who survived the entire hospital admission | | | | | |

## Association of ageing with biomarker concentrations after correcting for baseline disease severity

Supplementary Table S4: Differences in biomarker concentrations between age decades after correcting for baseline disease severity

|  | **ANCOVA  p-value*** |
| --- | --- |
| **Systemic inflammation and cytokine release** | |
| **CRP (mg/L)** | 0.019 |
| **MMP-8 (ng/mL)** | 0.152 |
| **TIMP1 (ng/mL)** | 0.817 |
| **IL-6 (pg/mL)** | 0.152 |
| **IL-8 (pg/mL)** | 0.325 |
| **IL-10 (pg/mL)** | 0.004 |
| **Endothelial cell activation and function** | |
| **sE-selectin (ng/mL)** | 0.003 |
| **sICAM-1 (ng/mL)** | 0.007 |
| **Fractalkine (pg/mL)** | 0.001 |
| **Angiopoietin-1 (ng/mL)** | 0.011 |
| **Angiopoietin-2 (ng/mL)** | 0.080 |
| **Angiopoietin-2/1 ratio** | 0.021 |
| **Coagulation activation** | |
| **Platelet count (x 10^9/L)** | <0.001 |
| **PT (sec)** | 0.627 |
| **D-Dimer (microg/mL)** | 0.525 |
| **Antitrombin (ng/mL)** | 0.750 |
| **Protein C (ng/mL)** | 0.344 |
| Abbreviations: ANCOVA: analysis of covariance, APACHE IV APS: Acute Physiology and Chronic Health Evaluation IV Acute Physiology score, SOFA: Sequential Organ Failure Score, CRP: C-reactive protein, MMP-8: matrix metalloproteinase-8, TIMP-1: tissue inhibitor of metalloproteinase 1, IL-6: interleukin 6, IL-8: interleukin 8, IL-10: interleukin 10, sE-selectin: soluble E-Selectin, sICAM: soluble intercellular adhesion molecule 1, PT: Prothrombin time.  The severity-adjusted ANCOVA included the APACHE IV APS, SOFA score, and presence of shock and acute kidney injury on admission as covariates.* All p-values are multiple testing corrected using the Benjamini-Hochberg procedure for the conduction of 17 ANCOVA’s (testing of 17 biomarkers). | |

## Sensitivity analysis

Supplementary Table S5: Sensitivity analysis of biomarkers of critically ill sepsis patients incorporating age as a continuous variable of which the methods depended on the linearity of the age with the analysed biomarker after box-cox transformation

|  | **Linearity of age with  biomarker *** | **p-value age coefficient linear  regression analysis** † | **p-value age coefficient  spline regression analysis** ‡ |
| --- | --- | --- | --- |
| **Systemic inflammation and cytokine release** | | |  |
| **CRP (mg/L)** | No | - | 0.080 |
| **MMP-8 (ng/mL)** | Yes | 0.374 | - |
| **TIMP1 (ng/mL)** | Yes | 0.374 | - |
| **IL-6 (pg/mL)** | Yes | 0.899 | - |
| **IL-8 (pg/mL)** | Yes | 0.404 | - |
| **IL-10 (pg/mL)** | Yes | 0.268 | - |
| **Endothelial cell activation and function** | | |  |
| **sE-selectin (ng/mL)** | Yes | 0.005 | - |
| **sICAM-1 (ng/mL)** | No | - | 0.002 |
| **Fractalkine (pg/mL)** | Yes | 0.002 | - |
| **Angiopoietin-1 (ng/mL)** | Yes | 0.004 | - |
| **Angiopoietin-2 (ng/mL)** | Borderline | 0.875 | 0.313 |
| **Angiopoietin-2/1 ratio** | Borderline | 0.167 | 0.157 |
| **Coagulation activation** | | |  |
| **Platelet count (x 10^9/L)** | Yes | 0.002 | - |
| **PT (sec)** | Yes | 0.404 | - |
| **D-Dimer (microg/mL)** | Yes | 0.070 | - |
| **Antitrombin (ng/mL)** | Yes | 0.493 | - |
| **Protein C (ng/mL)** | Yes | 0.051 | - |
| Abbreviations: CRP: C-reactive protein, MMP-8: matrix metalloproteinase-8, TIMP-1: tissue inhibitor of metalloproteinase 1, IL-6: interleukin 6, IL-8: interleukin 8, IL-10: interleukin 10, sE-selectin: soluble E-Selectin, sICAM: soluble intercellular adhesion molecule 1, PT: Prothrombin time * after boxcox transformation of the biomarker † p-value is calculated based on the coefficient of age in a linear regression analysis followed by a Benjamini-Hochberg correction for multiple testing for the conduction of 17 regressions ‡ p-value is calculated based on the total contribution (including all terms) of age modelled as a restricted cubic spline using 3 internal knots  followed by a Benjamini-Hochberg correction to correct for multiple testing for the conduction of 17 regressions (testing of 17 biomarkers). | | | |

## Demographics and clinical outcomes of whole blood transcriptome cohort

Supplementary Table S6: Baseline characteristics of critically ill sepsis patients (whole blood transcriptome cohort)

|  | **<50 years** | | **≥50 - <60 years** | **≥60 - <70 years** | **≥70 years** | **p-value** |
| --- | --- | --- | --- | --- | --- | --- |
| **n** | 88 | | 79 | 153 | 168 |  |
| **Demographics** |  | |  |  |  |  |
| **Age years, median [IQR]** | 39.50 [30.00, 47.00] | | 55.00 [53.00, 57.50] | 64.00 [62.00, 67.00] | 75.00 [72.00, 79.00] | <0.001 |
| **Male sex, n (%)** | 44 (50.0) | | 47 (59.5) | 102 (66.7) | 96 (57.1) | 0.075 |
| **White race, n (%)** | 61 (70.1) | | 68 (86.1) | 140 (92.1) | 159 (95.2) | <0.001 |
| **BMI, median [IQR]** | 22.86 [21.02, 25.55] | | 24.84 [21.56, 27.70] | 25.39 [22.84, 28.83] | 25.15 [22.86, 27.76] | <0.001 |
| **Medical admission, n (%)** | 64 (72.7) | | 48 (60.8) | 114 (74.5) | 125 (74.4) | 0.115 |
| **Comorbidity** |  | |  |  |  |  |
| **Charlson score *, median [IQR]** | 0.00 [0.00, 2.00] | | 2.00 [0.00, 3.50] | 2.00 [0.00, 4.00] | 2.00 [0.00, 3.00] | 0.006 |
| **Cardiovascular, n (%)** | 9 (10.2) | | 16 (20.3) | 34 (22.2) | 45 (26.8) | 0.023 |
| **Respiratory insufficiency, n (%)** | 7 (8.0) | | 2 (2.5) | 10 (6.5) | 13 (7.7) | 0.431 |
| **Hypertension, n (%)** | 14 (15.9) | | 25 (31.6) | 47 (30.7) | 66 (39.3) | 0.002 |
| **Diabetes, n (%)** | 7 (8.0) | | 12 (15.2) | 35 (22.9) | 42 (25.0) | 0.005 |
| **Malignancy, n (%)** | 14 (15.9) | | 24 (30.4) | 40 (26.1) | 47 (28.0) | 0.121 |
| **Renal disease, n (%)** | 10 (11.4) | | 16 (20.3) | 27 (17.6) | 27 (16.1) | 0.445 |
| **Immunocompromised, n (%)** | 33 (37.5) | | 23 (29.1) | 38 (24.8) | 21 (12.5) | <0.001 |
| **Chronic medication, n (%)** |  | |  |  |  |  |
| **Anticoagulants** | 8 (9.1) | | 11 (13.9) | 25 (16.3) | 29 (17.3) | 0.337 |
| **Antiplatelet drugs** | 4 (5.0) | | 13 (17.1) | 34 (24.1) | 70 (43.2) | <0.001 |
| **Site of infection, n (%)** |  | |  |  |  |  |
| **Cardiovascular** | 5 (5.7) | | 4 (5.1) | 3 (2.0) | 7 (4.2) | 0.457 |
| **Pulmonary** | 34 (38.6) | | 22 (27.8) | 65 (42.5) | 67 (39.9) | 0.176 |
| **Urinary** | 4 (4.5) | | 3 (3.8) | 15 (9.8) | 9 (5.4) | 0.196 |
| **Skin** | 4 (4.5) | | 8 (10.1) | 4 (2.6) | 2 (1.2) | 0.005 |
| **Abdominal** | 16 (18.2) | | 16 (20.3) | 25 (16.3) | 31 (18.5) | 0.901 |
| **Central nervous system** | 3 (3.4) | | 0 (0.0) | 4 (2.6) | 1 (0.6) | 0.167 |
| **Other infection site** † | 3 (3.4) | | 5 (6.3) | 6 (3.9) | 7 (4.2) | 0.796 |
| **Mixed infection** | 18 (20.5) | | 22 (27.8) | 31 (20.3) | 43 (25.6) | 0.459 |
| **Unknown site** ‡ | 3 (3.4) | | 1 (1.3) | 1 (0.7) | 4 (2.4) | 0.423 |
| **Causative pathogen primary site of infection** §**, n (%)** | |  |  |  |  |  |
| **Gram-positive bacteria** | 38 (43.2) | | 34 (43.0) | 64 (41.8) | 69 (41.1) | 0.986 |
| **Gram-negative bacteria** | 34 (38.6) | | 41 (51.9) | 71 (46.4) | 80 (47.6) | 0.363 |
| **Fungi** | 15 (17.0) | | 10 (12.7) | 25 (16.3) | 15 (8.9) | 0.164 |
| **Virus** | 5 (5.7) | | 4 (5.1) | 6 (3.9) | 4 (2.4) | 0.558 |
| **Other** | 9 (10.2) | | 3 (3.8) | 6 (3.9) | 5 (3.0) | 0.059 |
| **Unknown** | 14 (15.9) | | 9 (11.4) | 26 (17.0) | 33 (19.6) | 0.444 |
| **Disease severity on admission** | | | | | | |
| **APACHE IV APS, median [IQR]** | 69.00 [50.00, 92.25] | | 70.00 [54.50, 90.50] | 67.00 [51.00, 86.00] | 69.00 [54.75, 85.00] | 0.688 |
| **SOFA score** ll**, median [IQR]** | 7.00 [4.00, 9.25] | | 8.00 [6.00, 10.00] | 7.00 [4.00, 9.00] | 7.00 [5.00, 9.25] | 0.376 |
| **Shock, n (%)** | 22 (25.0) | | 31 (39.2) | 36 (23.5) | 53 (31.5) | 0.059 |
| **ARDS, n (%)** | 32 (36.4) | | 27 (34.2) | 42 (27.5) | 53 (31.5) | 0.496 |
| **Acute kidney injury, n (%)** | 30 (34.1) | | 35 (44.3) | 46 (30.1) | 81 (48.2) | 0.005 |
| Abbreviations: BMI: Body Mass Index, APACHE IV APS: Acute Physiology and Chronic Health Evaluation IV Acute Physiology score, SOFA: Sequential Organ Failure Score, ARDS: acute respiratory distress syndrome.  * The Charlson score was calculated without the age component † Other infection sites consisted of infections of bones and joints, the reproductive tract, mediastinum, the ear, throat or mouth.  ‡ Unknown site of infection consisted of infections of unknown source, systemic viral infections, and primary bacteremia. § Causative organisms of the primary site of infection do not add up to 100% as some patients suffered from multiple pathogens at the primary site.  ll The SOFA score was calculated without the Central Nervous System component | | | | | | |

Supplementary Table S7: Clinical outcomes of critically ill sepsis patients (whole blood transcriptome cohort)

|  | **<50 years** | **≥50 - <60 years** | **≥60 - <70 years** | **≥70 years** | **p-value** |
| --- | --- | --- | --- | --- | --- |
| n | 88 | 79 | 153 | 168 |  |
| **ICU-acquired complications, n (%)** | | | | |  |
| **Shock** | 10 (11.4) | 12 (15.2) | 33 (21.6) | 29 (17.3) | 0.224 |
| **ARDS** | 4 (4.5) | 6 (7.6) | 8 (5.2) | 11 (6.5) | 0.815 |
| **Acute kidney injury** | 3 (3.4) | 8 (10.1) | 24 (15.7) | 11 (6.5) | 0.006 |
| **ICU-acquired infections** | 10 (11.4) | 11 (13.9) | 18 (11.8) | 23 (13.7) | 0.915 |
| **Length of stay, median [IQR]** | | | | | |
| **ICU stay, days *** | 4.88 [1.63, 11.03] | 6.15 [3.45, 12.28] | 6.43 [2.33, 9.92] | 4.80 [2.11, 10.39] | 0.294 |
| **Hospital stay, days †** | 24.40 [10.79, 49.70] | 32.42 [13.46, 54.02] | 21.37 [14.61, 46.36] | 19.25 [10.87, 39.16] | 0.219 |
| **Mortality, n (%)** |  |  |  |  |  |
| **ICU** | 11 (12.5) | 19 (24.1) | 22 (14.4) | 44 (26.2) | 0.011 |
| **Hospital** | 17 (19.3) | 26 (32.9) | 43 (28.1) | 69 (41.1) | 0.003 |
| **Day 30** | 16 (18.2) | 24 (30.4) | 33 (21.6) | 61 (36.3) | 0.004 |
| **Day 60** | 22 (25.0) | 27 (34.2) | 43 (28.1) | 75 (44.6) | 0.003 |
| **Day 90** | 27 (30.7) | 31 (39.2) | 49 (32.0) | 81 (48.2) | 0.009 |
| Abbreviations: ARDS: acute respiratory distress syndrome, ICU: intensive care. * Length of ICU stay was only calculated in those who survived the entire ICU admission  † Length of hospital was only calculated in those who survived the entire hospital admission | | | | | |

## References

1. Charlson ME, Pompei P, Ales KL, MacKenzie CR. A new method of classifying prognostic comorbidity in longitudinal studies: development and validation. J Chronic Dis. England; 1987;40:373–83.

2. Bellomo R, Ronco C, Kellum JA, Mehta RL, Palevsky P. Acute renal failure - definition, outcome measures, animal models, fluid therapy and information technology needs: the Second International Consensus Conference of the Acute Dialysis Quality Initiative (ADQI) Group. Crit Care. 2004;8:R204-12.

3. Bernard GR, Artigas A, Brigham KL, Carlet J, Falke K, Hudson L, et al. The American-European Consensus Conference on ARDS. Definitions, mechanisms, relevant outcomes, and clinical trial coordination. Am J Respir Crit Care Med [Internet]. American Thoracic Society - AJRCCM; 1994;149:818–24. Available from: https://doi.org/10.1164/ajrccm.149.3.7509706

4. Klein Klouwenberg PMC, Ong DSY, Bos LDJ, de Beer FM, van Hooijdonk RTM, Huson MA, et al. Interobserver agreement of Centers for Disease Control and Prevention criteria for classifying infections in critically ill patients. Crit Care Med. United States; 2013;41:2373–8.

5. Scicluna BP, Klein Klouwenberg PMC, van Vught LA, Wiewel MA, Ong DSY, Zwinderman AH, et al. A Molecular Biomarker to Diagnose Community-acquired Pneumonia on Intensive Care Unit Admission. Am J Respir Crit Care Med [Internet]. American Thoracic Society - AJRCCM; 2015;192:826–35. Available from: https://doi.org/10.1164/rccm.201502-0355OC

6. Martin GS, Mannino DM, Moss M. The effect of age on the development and outcome of adult sepsis. Crit Care Med. United States; 2006;34:15–21.

7. Pierrakos C, Velissaris D, Bisdorff M, Marshall JC, Vincent J-L. Biomarkers of sepsis: time for a reappraisal. Crit Care. 2020;24:287.

8. Langfelder P, Horvath S. WGCNA: an R package for weighted correlation network analysis. BMC Bioinformatics [Internet]. 2008;9:559. Available from: https://doi.org/10.1186/1471-2105-9-559

9. Davenport EE, Burnham KL, Radhakrishnan J, Humburg P, Hutton P, Mills TC, et al. Genomic landscape of the individual host response and outcomes in sepsis: a prospective cohort study. Lancet Respir Med. 2016;4:259–71.

10. Li Z, Li Y, Wang X, Yang Q. PPP2R2B downregulation is associated with immune evasion and predicts poor clinical outcomes in triple-negative breast cancer. Cancer Cell Int [Internet]. 2021;21:13. Available from: https://doi.org/10.1186/s12935-020-01707-9

11. Horvath S, Dong J. Geometric Interpretation of Gene Coexpression Network Analysis. PLOS Comput Biol [Internet]. Public Library of Science; 2008;4:e1000117. Available from: https://doi.org/10.1371/journal.pcbi.1000117

12. Lou Y, Tian G-Y, Song Y, Liu Y-L, Chen Y-D, Shi J-P, et al. Characterization of transcriptional modules related to fibrosing-NAFLD progression. Sci Rep [Internet]. 2017;7:4748. Available from: https://doi.org/10.1038/s41598-017-05044-2

13. Raudvere U, Kolberg L, Kuzmin I, Arak T, Adler P, Peterson H, et al. g:Profiler: a web server for functional enrichment analysis and conversions of gene lists (2019 update). Nucleic Acids Res. 2019;47:W191–8.

14. Consortium GO. The Gene Ontology (GO) database and informatics resource. Nucleic Acids Res [Internet]. 2004;32:D258–61. Available from: https://doi.org/10.1093/nar/gkh036

15. Jassal B, Matthews L, Viteri G, Gong C, Lorente P, Fabregat A, et al. The reactome pathway knowledgebase. Nucleic Acids Res [Internet]. Oxford University Press; 2020;48:D498–503. Available from: https://pubmed.ncbi.nlm.nih.gov/31691815

16. Chin C-H, Chen S-H, Wu H-H, Ho C-W, Ko M-T, Lin C-Y. cytoHubba: identifying hub objects and sub-networks from complex interactome. BMC Syst Biol [Internet]. 2014;8:S11. Available from: https://doi.org/10.1186/1752-0509-8-S4-S11

17. Hofman A, Breteler MMB, van Duijn CM, Krestin GP, Pols HA, Stricker BHC, et al. The Rotterdam Study: objectives and design update. Eur J Epidemiol [Internet]. 2007/10/23. Springer Netherlands; 2007;22:819–29. Available from: https://pubmed.ncbi.nlm.nih.gov/17955331
