# Supplementary material for: Association between age and the host response in critically ill patients with sepsis
Source: Crit Care. 2022 Dec 13;26:385. doi: 10.1186/s13054-022-04266-9 (PMC9747080; doi:10.1186/s13054-022-04266-9)
Supplement: Supplementary file 2 — Additional file 2: Fig. S1. Flowchart MARS cohort. Fig. S2. Hierarchical average linkage clustering to detect outlier samples. Fig. S3. Weighted gene co-expression network analysis (WGCNA) network construction. Fig. S4. Weighted gene co-expression network analysis cluster dendogram and cluster assignment. Fig. S5. Top 5 significantly different expressed pathways per pathway database of the turquoise, green, pink and brown modules. [file 13054_2022_4266_MOESM2_ESM.docx]

# **Additional file 2 Supplementary Figures**

# **Association between age and the host response in critically ill patients with sepsis**

Authors
Erik H.A. Michels, Joe M. Butler, Tom D.Y. Reijnders, Olaf L. Cremer, Brendon P. Scicluna, Fabrice Uhel, Hessel Peters-Sengers, Marcus J. Schultz, Julian C. Knight, Lonneke A. van Vught, Tom van der Poll, on behalf of the MARS consortium

# **List of supplementary figures**

Supplementary Figure S1: Flowchart MARS cohort................................................................2
Supplementary Figure S2: Hierarchical average linkage clustering to detect
outlier samples........................................................................................................................3

Supplementary Figure S3: Weighted gene co-expression network analysis (WGCNA) network construction............................................................................................................................4
Supplementary Figure S4: Weighted gene co-expression network analysis cluster dendogram and cluster assignment...........................................................................................................5
Supplementary Figure S5: Top 5 significantly different expressed pathways per pathway database of the turquoise, green, pink and brown modules..................................................6

**Figure S1:** Flowchart MARS cohort
**Description Figure S1:** Abbreviations:
Jan: January, ICU: intensive care unit.

Age on admission
<50
n=421
(22%)

Age on admission
≥70
n=280
(45%)

Age on admission
≥60 - <70
n=259
(46%)

Age on admission
<50
n=187
(44%)

Age on admission
≥50 - <60
n=163
(44%)

Age on admission
≥50 - <60
n=79
(21%)

Age on admission
≥60 - <70
n=153
(28%)

Whole blood transcriptome cohort
(% of clinical cohort)

Host response biomarker cohort
(% of clinical cohort)

Age on admission
≥70
n=168
(27%)

Age on admission
<50
n=88
(21%)

Clinical cohort

(% of sepsis admissions)

Age on admission
≥70
n=618
(32%)

Age on admission
≥60 - <70

n=545
(28%)

Age on admission
≥50 - <60

n=368
(19%)

**Excluded**
- Transfer from another ICU, n=296

- Readmissions, n=537

Unique sepsis admissions
n=1952

Sepsis admissions

Jan 2011 – Jan 2014
n=2785

**Figure S2** Hierarchical average linkage clustering to detect outlier samples

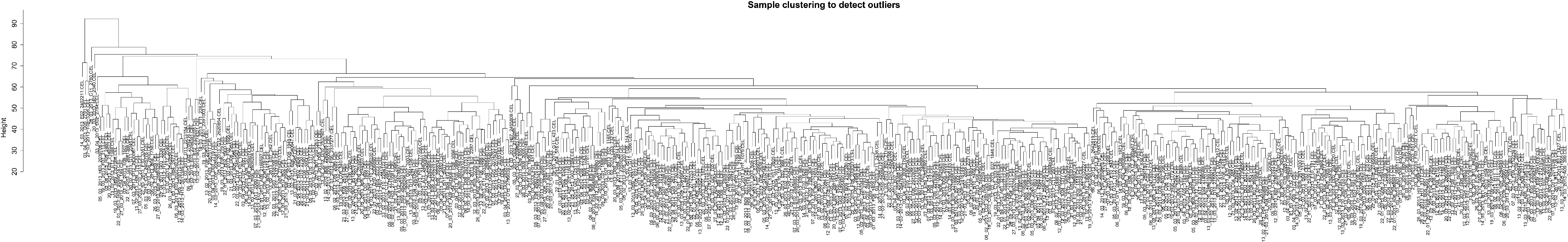


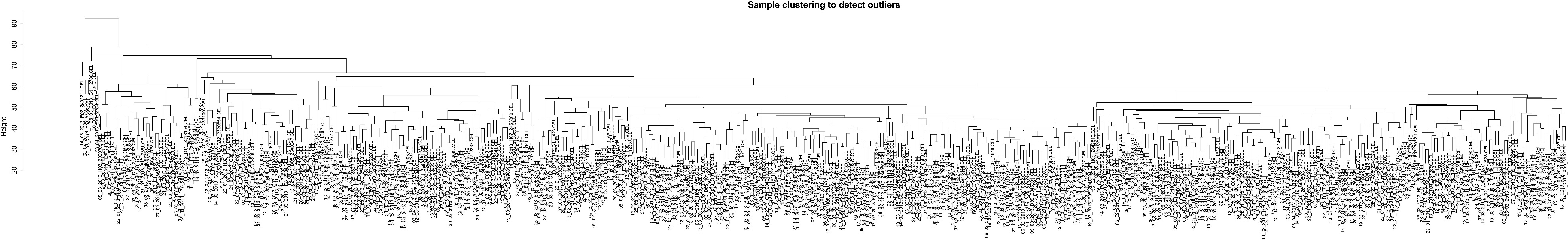

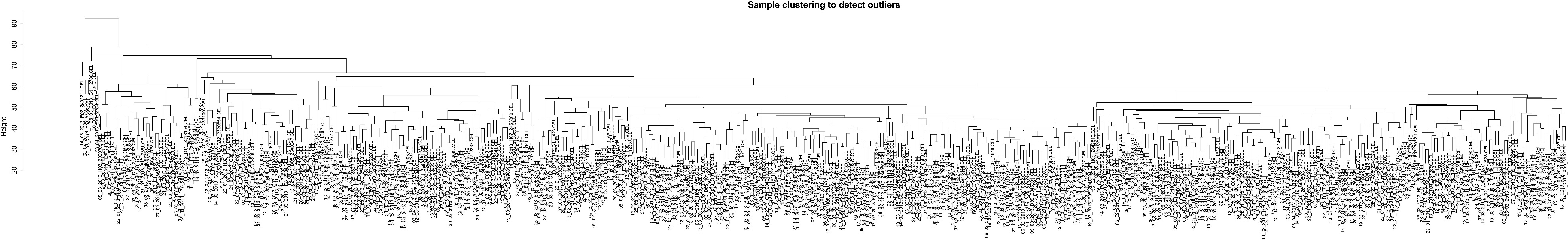

**Description Figure S2:** Hierarchical average linkage clustering of 488 patients. The upper panel resembles the left side and the lower panel resembles the right side of the dendogram.

**Figure S3:** Weighted gene co-expression network analysis (WGCNA) network construction


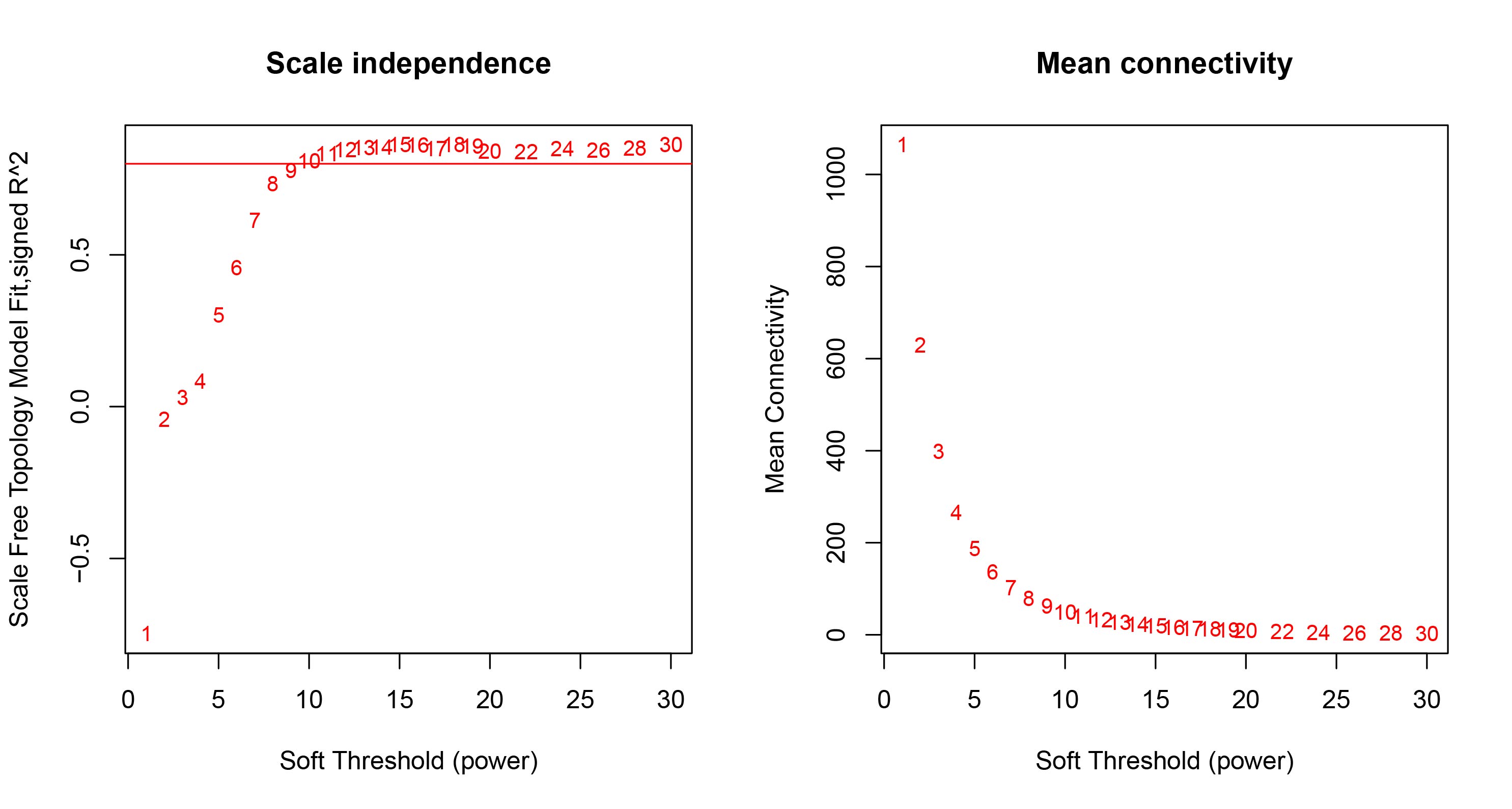
 **Description Figure S3:** The left panel shows the Scale independence by evaluating the scale-free fit index (signed R^2^ for a scale free network) of several Soft Thresholds (powers). The red line indicates a R^2^ of 0.8. The right panel shows the Mean connectivity of several powers. A power of 13 showed to be ideal. At this power value, a high R^2^ (close to 0.9) in combination of flattening of the mean connectivity curve is observed.

**Figure S4:** Weighted gene co-expression network analysis cluster dendogram and cluster assignment


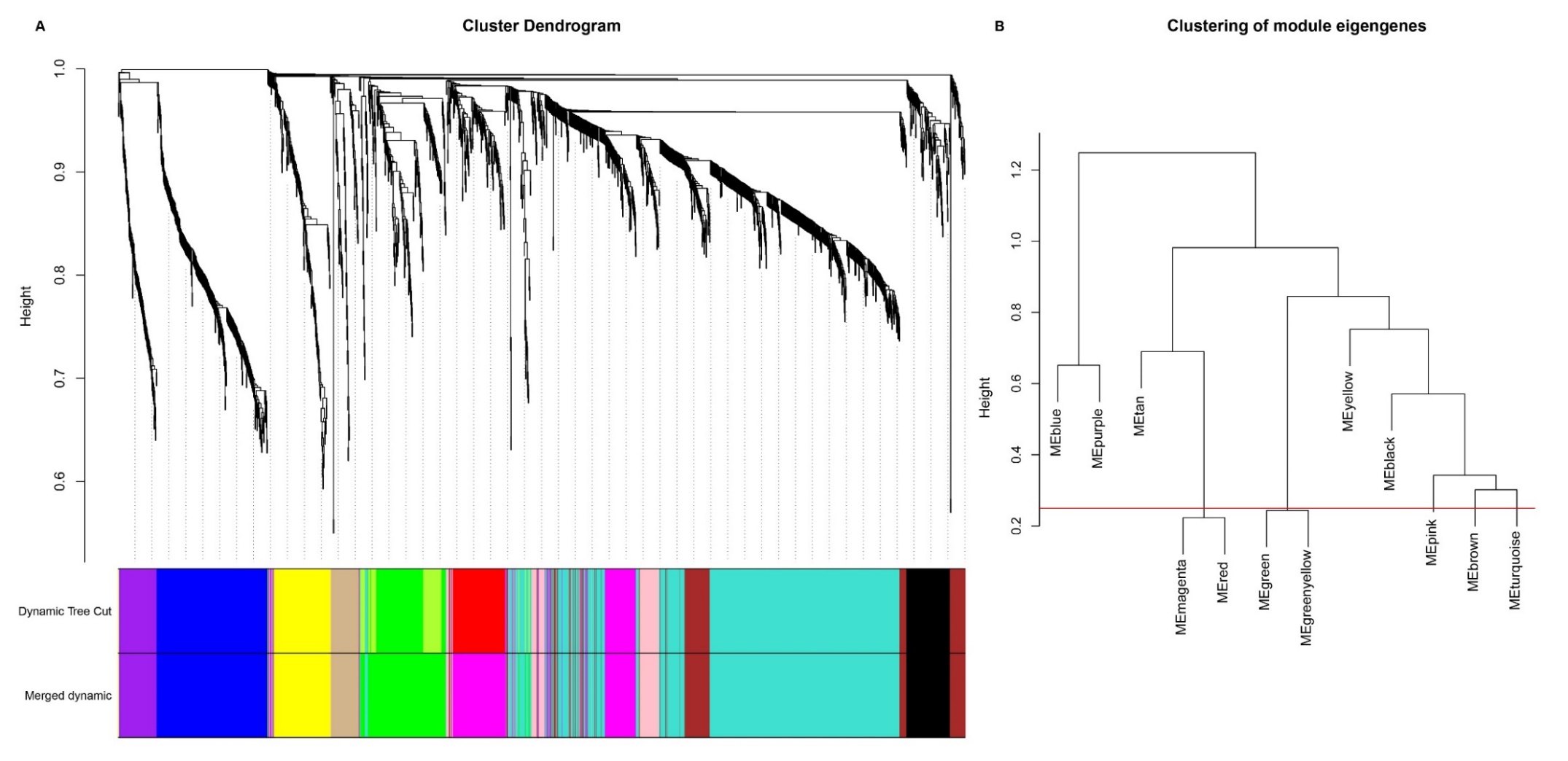
**Description Figure S4: A)** Cluster Dendogram and assigned modules. Clustering of genes was based on dissimilarity (1 – values of the topological overlap matrix). The minimal cluster size was set to 40 genes. Branches characterise groups of highly correlated genes which are represented by colours referred to as modules. Clustering of 1947 genes of 488 patients resulted in 12 modules. The colours within the Dynamic Tree Cut bar plot represent the modules before merging based on similarity. The colours in the Merged dynamic colours barplot represent the modules after merging. **B)** Clustering of module eigengenes. Modules with a correlation coefficient greater than 0.75 were merged. The magenta and red module were merged into the magenta module and the green and green yellow module into the green module. After merging, 10 modules remained.

**
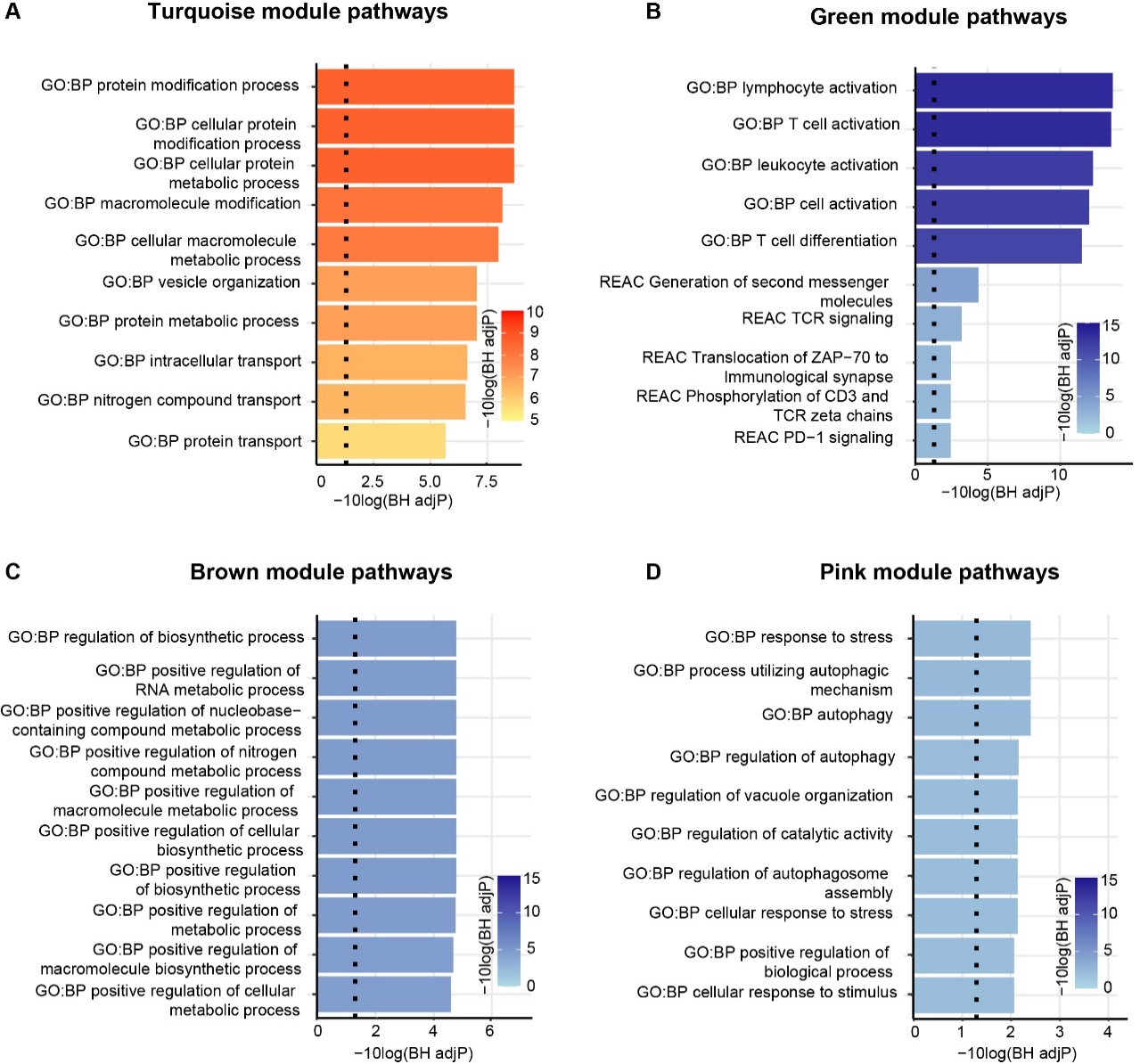
Figure S5:** Top 5 significantly different expressed pathways per pathway database of the turquoise, green, pink and brown modules

**Description Figure S5:** Top 5 pathways per pathway database (the Gene Ontology Biological Process (GO:BP) and Reactome database (REAC) of all genes within the module (independent of the edge). If none of the Reactome pathways were significant, 10 Gene Ontology Biological Process pathways were displayed. A) Turquoise module pathway analysis. B) Green module pathway analysis. C) Brown module pathway analysis. D) Pink module pathway analysis.
